# Protease Activated Probes for Real-Time Ratiometric Imaging of Solid Tumors

**DOI:** 10.1021/acscentsci.3c00261

**Published:** 2023-05-04

**Authors:** Franco F. Faucher, Kevin J. Liu, Emily D. Cosco, John C. Widen, Jonathan Sorger, Matteo Guerra, Matthew Bogyo

**Affiliations:** † Department of Chemistry, Stanford University, Stanford, California 94305, United States; ‡ Program in Cancer Biology, Stanford University School of Medicine, Stanford, California 94305 United States; § Department of Pathology, Stanford University School of Medicine, Stanford, California 94305, United States; ⊥ Intuitive Surgical Inc., Sunnyvale, California 94086, United States; # Department of Chemical and Systems Biology, Stanford University School of Medicine, Stanford, California 94305, United States; ∥ Department of Microbiology and Immunology, Stanford University School of Medicine, Stanford, California 94305, United States

## Abstract

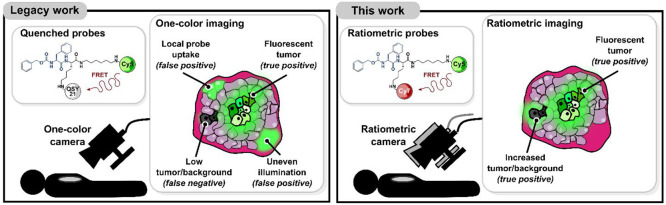

Surgery is the preferred treatment option for most solid tumors. However, inaccurate detection of cancer borders leads to either incomplete removal of malignant cells or excess excision of healthy tissue. While fluorescent contrast agents and imaging systems improve tumor visualization, they can suffer from low signal-to-background and are prone to technical artifacts. Ratiometric imaging has the potential to eliminate many of these issues such as uneven probe distribution, tissue autofluorescence, and changes in positioning of the light source. Here, we describe a strategy to convert quenched fluorescent probes into ratiometric contrast agents. Conversion of the cathepsin-activated probe, 6QC-Cy5, into a two-fluorophore probe, 6QC-RATIO, significantly improved signal-to-background *in vitro* and in a mouse subcutaneous breast tumor model. Tumor detection sensitivity was further enhanced using a dual-substrate AND-gate ratiometric probe, Death-Cat-RATIO, that fluoresces only after orthogonal processing by multiple tumor-specific proteases. We also designed and built a modular camera system that was coupled to the FDA-approved da Vinci Xi robot, to enable real-time imaging of ratiometric signals at video frame rates compatible with surgical workflows. Our results demonstrate that ratiometric camera systems and imaging probes have the potential to be clinically implemented to improve surgical resection of many types of cancer.

## Abstract 2

This work introduces probes and a camera system that enable real-time ratiometric imaging of solid tumors in an intraoperative setting to overcome limitations of previous one dye systems.

## Introduction

Surgical resection is a primary treatment option for most types of solid tumors.^1,2^ The success of this procedure largely depends on the tactile and visual ability of the surgeon to correctly distinguish tumors from surrounding healthy tissue.^1,2^ Depending on the tumor type, stage, and localization, incomplete removal of a cancer mass occurs on average in 9% of procedures and can be as high as 21% and 35% in prostate and ovarian cancer, respectively.^3^ This inability to fully resect a tumor often triggers supplemental rounds of surgery, radiation, or chemotherapy, which in addition to translating into a worse prognosis and reduced quality of life, is also extremely costly to the healthcare system.^3^

One of the long-term goals of the molecular imaging field is to develop tools that provide imaging contrast with a molecular level of precision. Coupled with recent advances in the selectivity and sensitivity of cancer detection, the widespread adoption of molecular imaging methods has led to improved surgical outcomes and reduced risk of iatrogenic damage in patients.^4^ While technologies such as positron emission tomography (PET), magnetic resonance imaging (MRI), and computed tomography (CT) currently play important roles in diagnosis and preoperative planning, there remains a need to develop contrast agents and imaging modalities for intraoperative surgical guidance. Current intraoperative imaging strategies rely on the use of ultrasound, X-ray fluoroscopy, and fluorescent optical contrast agents.^5^ Ultrasound imaging has been shown to increase the proportion of clear resection margins by about 15% when compared to tumor detection via conventional methods such as palpation.^6^ However, techniques such as ultrasonography^5,7^ fail to detect superficial lesions and require continuous tissue contact that limits applications in many surgical workflows. Alternatively, X-ray fluoroscopy provides a satisfactory imaging frame rate and unparalleled imaging depth but exposes patients and healthcare professionals to harmful doses of radiation. In addition, its use in clinics has been decreasing steadily due to poor spatiotemporal resolution and lack of innovation in contrast agents and detection systems.^8^

Fluorescent optical contrast agents have optimal properties for use in surgical guidance. In addition to providing a safe alternative to X-ray fluoroscopy, fluorescent contrast enables sensitive visualization of superficial lesions without requiring direct tissue contact needed for ultrasound-guided surgery. Moreover, a depth of detection of ∼5 mm can be achieved when near-infrared (NIR) and shortwave-infrared dyes are used in combination with optimized camera systems,^4,5,9,10^ which is adequate for most intraoperative applications.^5^ Earlier this year, a decade-long effort in probe and imaging device development resulted in the FDA approval of the first targeted fluorescent optical contrast agent, Cytalux (pafolacianine), for use in ovarian cancer surgery.^11−13^ Cytalux is an affinity-based probe that binds selectively to folate receptor-expressing tumor cells. In 26.9% of the patients administered with Cytalux, fluorescent signals correctly identified lesions which would have otherwise been missed after visual inspection or palpation.^13^

**Figure 1:**
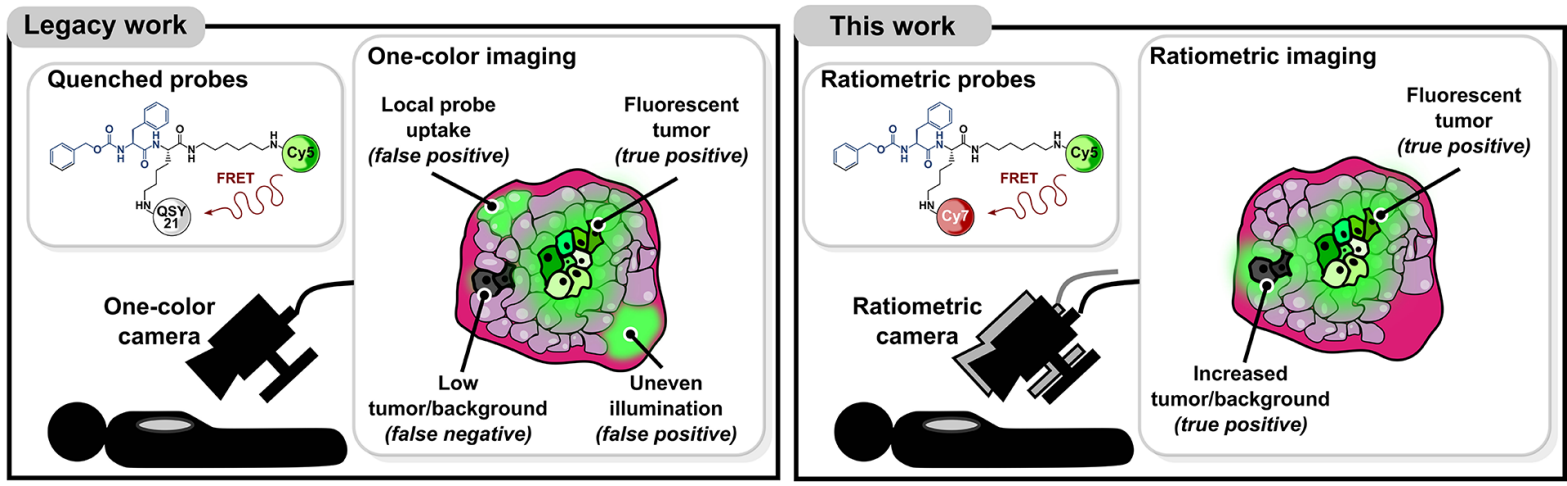
Fluorescently quenched reporters such as 6QC-Cy5 enable the detection of cancer lesions intraoperatively. However, one-dye imaging suffers from technical artifacts and poor signal-to-background ratios (left). This work introduces probes and a camera system that enable real time ratiometric imaging of solid tumors in an intraoperative setting (right).

As an alternative to affinity-based probes which are always fluorescent, activity-based optical contrast agents are “optically silent” until activated by tumor-specific enzymes to generate a NIR signal. This results in rapid generation of signal contrast with low overall backgrounds. In particular, several protease-activated fluorescent imaging probes have reached clinical studies.^14−22^ Examples include probes that are activated by matrix metallo proteases secreted by tumor cells^23^ as well as lysosomal cathepsin proteases secreted by tumor associated macrophages (TAMs).^24^ For many protease-activated imaging agents, a peptidic substrate scaffold links a dye-quencher pair in close proximity (<10 nm) enabling Förster resonance energy transfer (FRET) to occur. After entering the tumor microenvironment, the probe is proteolytically cleaved, resulting in displacement of the quencher and turn-on of the dye due to loss of energy transfer. Substrate probes such as LUM015,^14−17^ 6QC-Cy5,^24^ 6QC-NIR,^25^ and DEATH-CAT-NIR^26^ produce a signal upon cleavage of the substrate by a protease (Figure 1, left). The cleaved fluorescent fragment is retained within the tumor microenvironment by leveraging the latent lysosomotropic effect (LLE) in which protonation of the free amine reduces its release from lysosomal compartments.^24^ One of the main benefits of substrate probes is that the protease releases the substrate after cleavage, allowing it to cleave additional substrates, resulting in a signal amplification over time.^27,28^

Despite advancements in probe engineering and imaging systems,^27,29−31^ all classes of fluorescent-based contrast agents suffer from overall high background signals which can result from tissue autofluorescence, local changes in pH and ionic composition, as well as nonspecific probe distribution and accumulation.^7^ These background factors both reduce overall contrast and increase the rate of false positive and negative signals (Figure 1, left).^7^

Ratiometric imaging has the potential to overcome many of the primary limitations that presently hinder optical contrast agents (Figure 1, right). In ratiometric imaging, the change in emission intensity of two fluorophores is recorded simultaneously, and a ratio of the two signals is computed. The result is a self-normalized signal that greatly increases sensitivity and improves signal quantification.^32,33^ In addition with ratiometric imaging, measurement of one fluorophore of interest which is activated by a specific biochemical process (the “reporter”) can be normalized by the signal of a second reference fluorophore.^34^ In an optimally designed ratiometric imaging probe, the intensity increase of one dye is linked to a decrease in the signal of the other fluorophore. Therefore, the amplitude ratio of baseline to signal will be greater with respect to intensity relative to a single fluorophore quenched probe.^32,33^ Hence, ratiometric imaging amplifies the detected signals, a feature of great importance in intraoperative imaging, as it may improve the detection of small lesions that would otherwise be missed.

In this work, we demonstrate a general strategy to convert existing quenched fluorescent probes into ratiometric versions with improved imaging characteristics. Specifically, we synthesized ratiometric forms of the cathepsin substrate-probe 6QC^24^ as well as the AND-gate dual action cathepsin/caspase probe Death-Cat-2.^26^ By converting quenched fluorescent probes to ratiometric probes we found that it was possible to dramatically reduce background signals and increase sensitivity as well as achieve tumor to background ratios substantially higher than ratios reported for other optical contrast agents. In addition, we demonstrate that our ratiometric probes overcome common issues that limit fluorescence-guided surgery applications, including nonspecific probe accumulation and incorrect positioning of the camera. Finally, we present an imaging device that enables real-time ratiometric imaging of large as well as small metastasis-like tumors at frame rates that are compatible with clinical applications. By coupling the camera to the FDA-approved da Vinci Xi platform, we demonstrate the feasibility of ratiometric imaging-guided robotic surgery *in vivo*.

## Results

### *In Vitro* Characterization of Ratiometric Protease-Activated Probes

**Figure 2:**
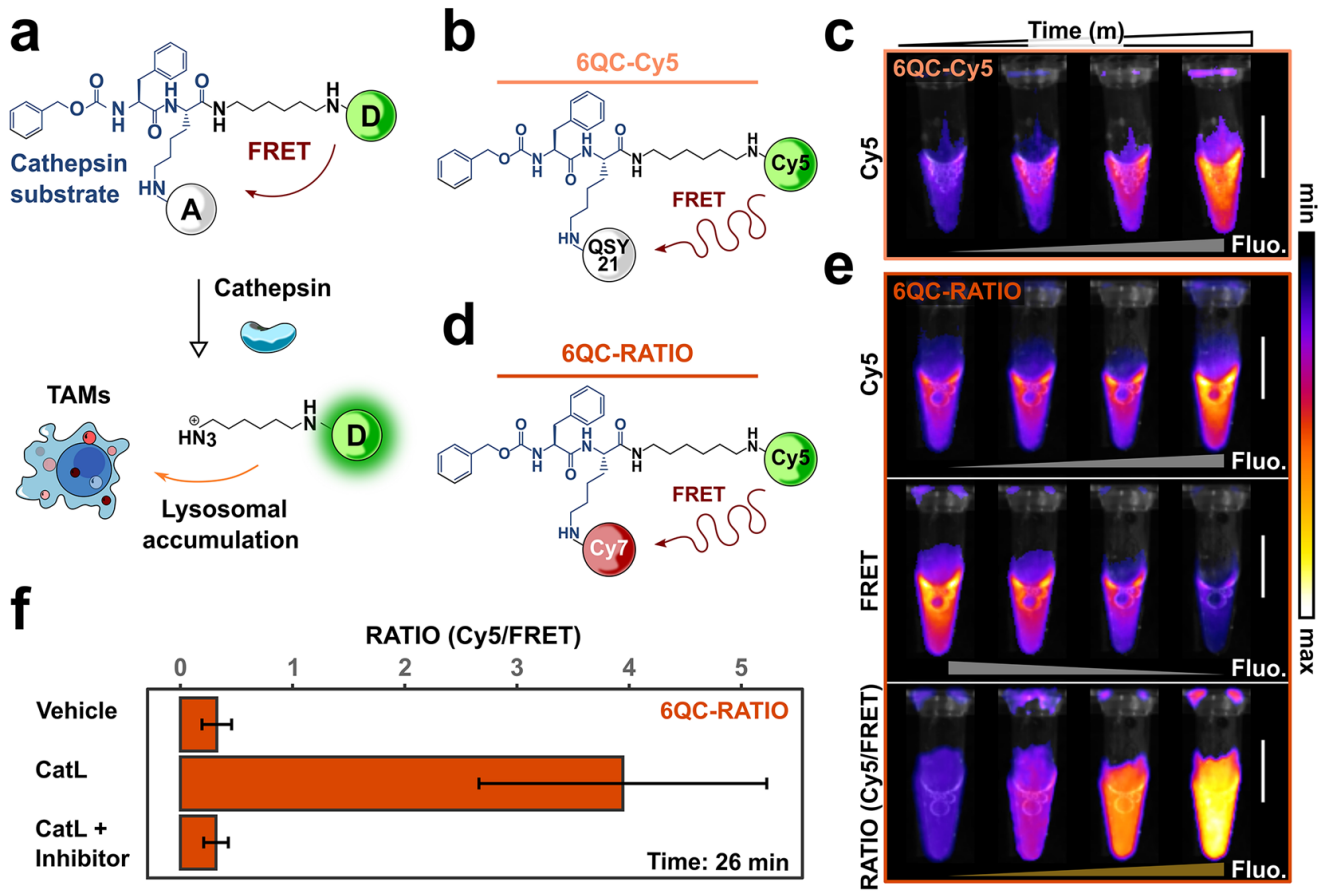
Structure and *in vitro* characterization of ratiometric optical contrast agents. (A) Schematic of 6QC probes. A peptidic substrate that is cleaved by cathepsins is flanked by an energy donor and an energy acceptor. Cathepsins in the tumor microenvironment cleave the peptide leading to donor fluorescence dequenching. The cleaved product localizes within lysosomes of tumor-associated macrophages enabling signal retention. D: energy donor, A: energy acceptor, TAMs: Tumor-associated macrophages. (B) Structure of 6QC-Cy5. (C) Time course imaging of vials containing 6QC-Cy5 [100 nM] after addition of cathepsin L [10 nM]. An overlay of the Cy5 channel and brightfield image is shown. (D) Structure of 6QC-RATIO. (E) Time course imaging of vials containing 6QC-RATIO [100 nM] after addition of cathepsin L [10 nM]. From top to bottom, images show an overlay of the Cy5 channel and brightfield image, an overlay of the FRET channel and brightfield image, and an overlay of the RATIO channel (generated by the division of the Cy5 over the FRET channels) and brightfield image. (F) Bar graph showing quantification of RATIO channel in vials containing 6QC-RATIO [100 nM], 26 min after addition of either vehicle, or cathepsin L [10 nM], or cathepsin L preincubated 30 min with a cathepsin inhibitor E64d [10 μM]. Data are shown as mean ± s.d. Time points shown correspond to 1, 5, 13, and 26 min after cathepsin L addition. Scale bars: 1 cm.

The fluorescently quenched probe, 6QC-Cy5 is an optical contrast agent that exploits a latent lysosomotropic effect for use in fluorescence-guided tumor surgery.^35^ Upon cleavage by cathepsins, it accumulates inside lysosomal compartments of tumor-associated macrophages that populate the tumor microenvironment (Figure 2a). The probe features a cathepsin cleavable sequence, *N*-benzyloxycarbonyl phenylalanyl lysine (zFK), and a FRET pair composed of sulfo-Cy5, serving as energy donor, and sulfo-QSY-21, a black hole quencher serving as energy acceptor (Figure 2b). In the uncleaved probe, Cy5 fluorescence is quenched due to energy transfer to QSY-21 (Figure 2c). The addition of human cathepsin results in cleavage of the amide bond between the lysine and the aliphatic linker causing an increase in Cy5 fluorescence due to subsequent loss of FRET (Figure 2c). The *in vitro* and *in vivo* visualization of current quenched^24^ and affinity probes^11^ as well as of the FDA-approved ICG dye^36^ is based on the detection of the fluorescence intensity and distribution of a single fluorophore. Here, we designed and synthesized a ratiometric optical contrast agent, 6QC-RATIO, by converting the dye-quencher pair of 6QC-Cy5 into a two-dye system composed of a Cy5 and a Cy7 FRET pair (Figure 2d and Supplemental Methods). Upon addition of cathepsin L, we detect an increase in the Cy5 channel and a decrease in the sensitized Cy7 (FRET) channel (Figure 2e). We then generated the ratiometric channel (RATIO) by pixel-to-pixel division of the donor and acceptor channels (Figure 2e). To test for signal specificity, we incubated 6QC-RATIO with either vehicle, cathepsin L, or cathepsin L pretreated with the specific inhibitor E64d ([10 μM] for 30 min). We detected an increase in the RATIO signal when vehicle treated cathepsin L was added to 6QC-RATIO and this signal was eliminated when the enzyme was inhibited by E64d (Figure 2f). Furthermore, we characterized FRET transfer by calculating the fold change in donor fluorescence emission upon cleavage by cathepsin L and found the changes to be dose dependent (Figure S1a and b). We also characterized the signal localization and selectivity of 6QC-RATIO in cells to assess the performance of the new reporter. We found that 6QC-RATIO localizes to lysosomes of RAW 264.7 (ATCC TIB-71) macrophages upon processing, with a significant colocalization between the 6QC-RATIO signal and a commercial lysosomal tracker (LysoTracker; Pearson correlation coefficient 0.57 ± 0.26, *n* = 210; Area overlap: 0.96 ± 0.02, *n* = 210; Figure S2a and b). In addition, we preincubated cells with E64d ([100 μM] for 90 min) which abolished Cy5 fluorescence in lysosomes (Figure S3a and b) confirming that 6QC-RATIO processing is cathepsin dependent.

### Use of Ratiometric Protease-Activated Probes Enhances Tumor to Background Signal *In Vivo*

**Figure 3:**
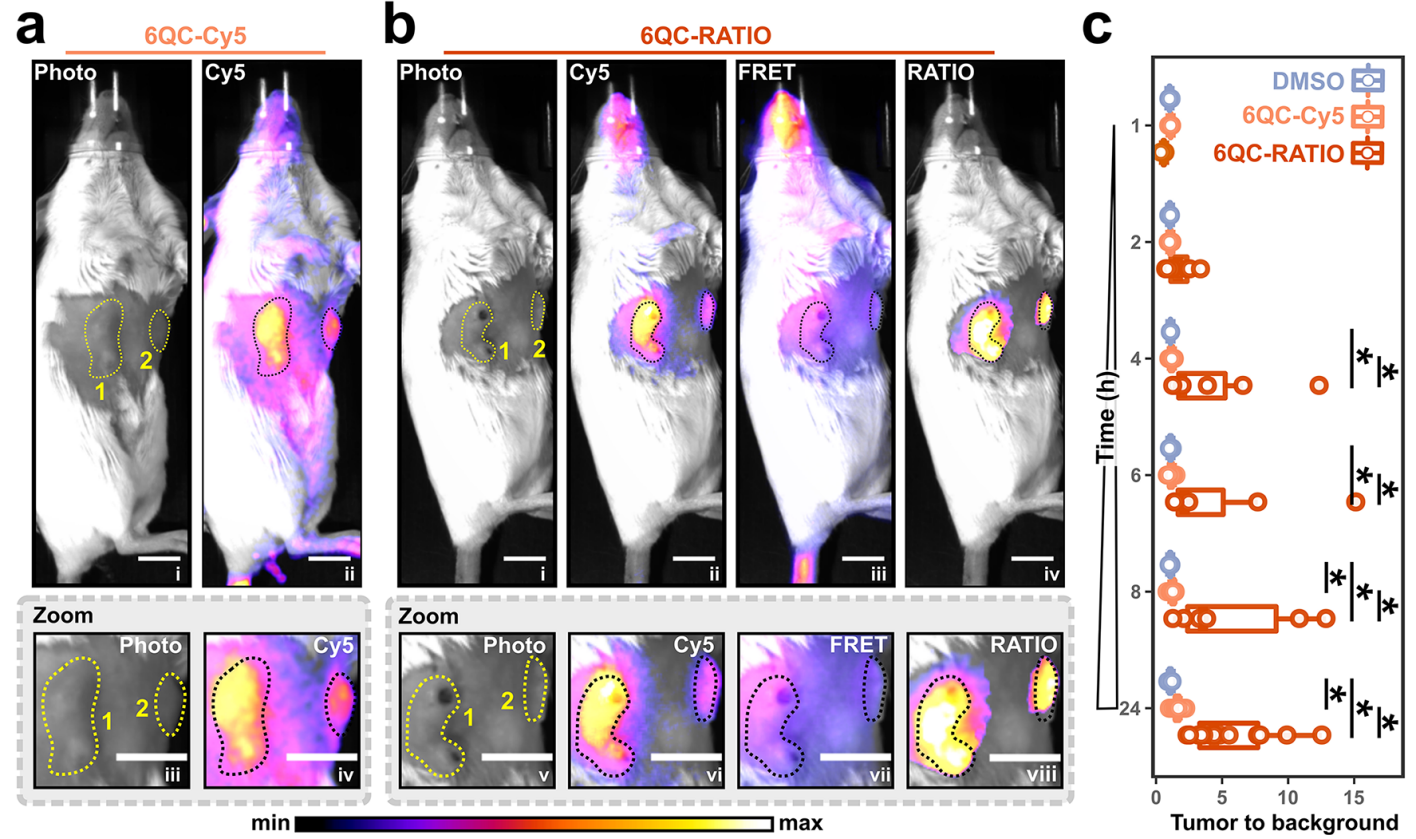
*In vivo* characterization of ratiometric optical contrast agents. (A) Representative fluorescence images showing a mouse with two breast tumors after injection with 6QC-Cy5 [10 nmol] 24 h prior to imaging. The left panel shows the brightfield image; the right panel shows the overlay between the Cy5 channel and the brightfield image. The zoom inlet shows a magnification of the two tumors. The tumor closer to the camera (area 1) is located in the eighth mammary fat pad; the tumor further to the camera (area 2) is located in the third mammary fat pad. (B) Representative fluorescence images showing a mouse with two breast tumors injected with 6QC-RATIO [10 nmol] 24 h prior to imaging. From left to right, the panels show the brightfield image, the overlay between the Cy5 channel and the brightfield image, the overlay between the FRET channel and the brightfield image, and the overlay between the RATIO channel and the brightfield image. The zoom inlet shows a magnification of the two tumors. The tumor closer to the camera (area 1) is located in the eighth mammary fat pad; the tumor further from the camera (area 2) is located in the third mammary fat pad. (C) Box and point plots showing the time-dependent quantification of the tumor to background signal in mice injected with either DMSO, 6QC-Cy5 [10 nmol] or 6QC-RATIO [10 nmol]. Tumor to background has been calculated from images acquired at the indicated time points. Points indicate single tumors. *N*: 3 to 9 mice per group, 3 to 18 tumors per condition. Statistics were calculated via Wilcoxon rank sum test, and an asterisk is indicated for statistically different means (*p* < 0.05) between groups. Scale bars: 1 cm.

To evaluate the performance of ratiometric optical contrast agents *in vivo*, we employed the 4T1 orthotopic breast cancer mouse model. We injected 4T1 (ATCC CRL-2539) mammary carcinoma cells subcutaneously into the third and eighth mammary fat pads. After seeding tumors for 7 to 10 days, we intravenously administered the optical contrast agent. Twenty-four hours after reporter injection, both probes generated a strong Cy5 signal localized in the tumor areas (Figure 3a and b, panel ii, areas 1 and 2). In addition to the Cy5 channel, we recorded 6QC-RATIO fluorescence in the FRET channel (Figure 3b, panels iii and vii) which enabled us to compute a ratiometric image (RATIO; Figure 3b, panels iv and viii). To demonstrate that the ratiometric signal is independent of camera and light source positioning with respect to the area of interest, we imaged tumors that were placed in a distal area with respect to the center of the field of view of the imaging device (Figure 3a and b, panel i, area 2). The probe was able to highlight the distal, out of focus, tumors (Figure 3b, panels iv and viii, area 2), while the one-dye (Cy5) signal did not (Figure 3a, panels ii and iv, area 2 and Figure 3b, panels ii and vi, area 2). Therefore, tumor tissue that would be otherwise confused with the background (Figure 3a and b, area 2) was clearly identifiable in the RATIO channel. To quantify the contrast produced by the ratiometric probe compared to 6QC-Cy5, we calculated the tumor to background ratio (TBR) signals in the 4T1 model. We calculated TBR values for the Cy5 channel for mice injected with 6QC-Cy5 and in the RATIO channel for 6QC-RATIO (Figure 3c). At 4 h post injection, the TBR of mice treated with 6QC-RATIO (TBR: 4.21 ± 4.0) was significantly higher than mice treated with DMSO (TBR: 1.09 ± 0.01) or 6QC-Cy5 (TBR: 1.18 ± 0.12) (Figure 3c). By 24 h post probe injection, the 6QC-RATIO signal reached an average TBR of 5.47 ± 3.03, compared to a TBR of 1.61 ± 0.3 for 6QC-Cy5, demonstrating that ratiometric imaging enhanced tumor contrast to the highest level reported for this class of optical contrast agents.

### Ratiometric Imaging with Logic-Gated Dual Substrate Probes

**Figure 4:**
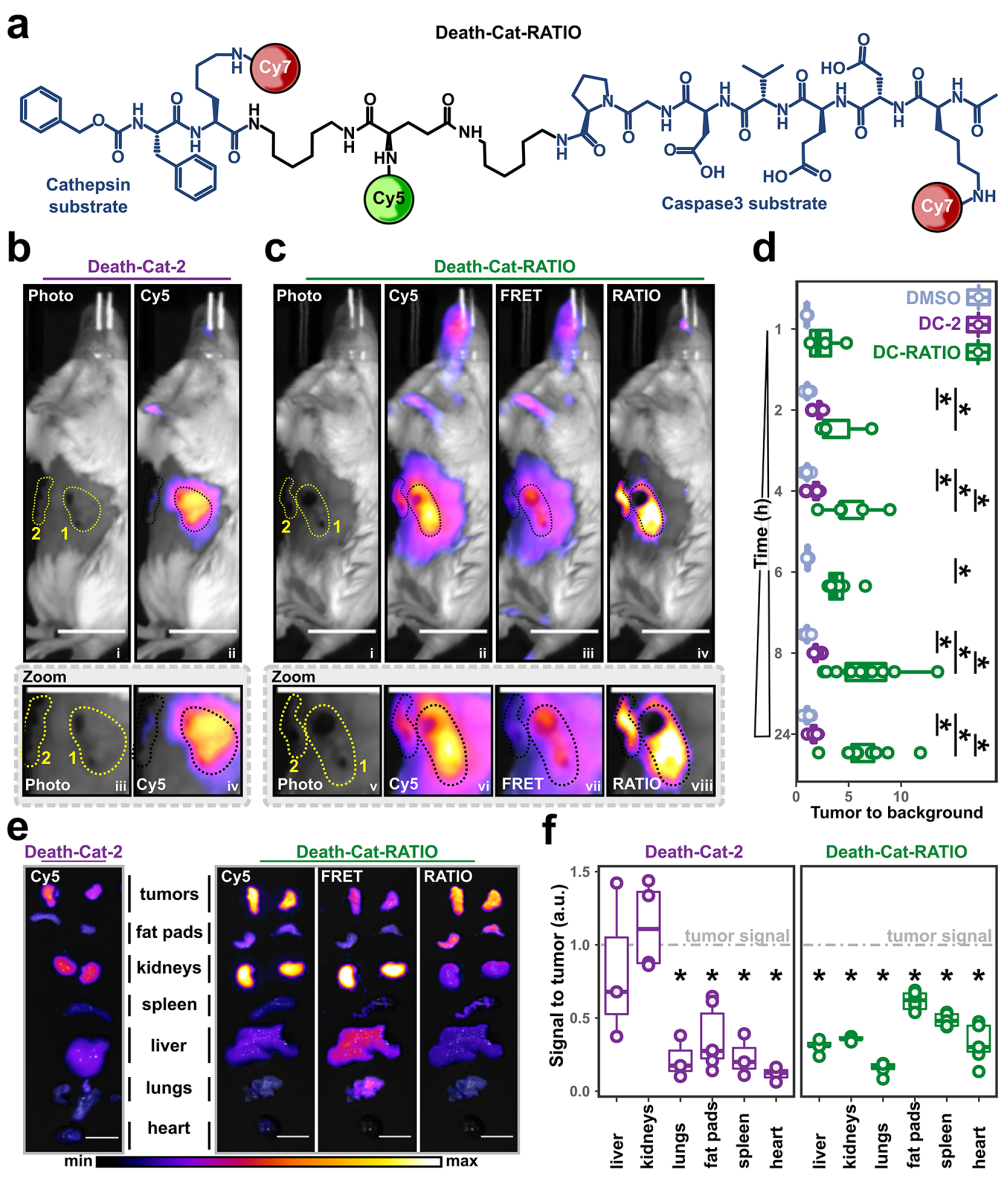
*In vivo* characterization of ratiometric logic-gated optical contrast agents. (a) Structure of Death-Cat-RATIO. (b) Representative fluorescence images showing a mouse with two breast tumors after injection with Death-Cat-2 [10 nmol] 24 h prior to imaging. The left panel shows the brightfield image; the right panel shows the overlay between the Cy5 channel and the brightfield image. The zoom inlet shows a magnification of the tumor. The tumor closer to the camera (area 1) is located in the third mammary fat pad; the tumor further from the camera (area 2) is located in the eighth mammary fat pad. (c) Representative fluorescence images showing a mouse with two breast tumors injected with Death-Cat-RATIO [10 nmol] 24 h prior to imaging. From left to right, the panels show the brightfield image, the overlay between the Cy5 channel and the brightfield image, the overlay between the FRET channel and the brightfield image, and the overlay between the RATIO channel and the brightfield image. The zoom inlet shows a magnification of the two tumors. The tumor closer to the camera (area 1) is located in the third mammary fat pad; the tumor further from the camera (area 2) is located in the 8th mammary fat pad. (d) Box and point plots showing the time-dependent quantification of the tumor to background signal in mice injected with either DMSO, Death-Cat-2 [10 nmol], or Death-Cat-RATIO [10 nmol]. Tumor to background has been calculated from images acquired at the indicated time points. (e) Representative images of organs excised from mice injected with either Death-Cat-2 [10 nmol] (left panel) or Death-Cat-RATIO [10 nmol] (right panel). (f) Box and point plot showing the fold change in signal in organs compared relative to tumors. Data are normalized to the Cy5 fluorescence for Death-Cat-2, and to the RATIO signal for Death-Cat-RATIO. In d and f, points indicate individual tumors. N: 3 to 6 mice per group, 3 to 12 tumors per condition, and 3 to 10 organs per condition. Statistics were calculated via Wilcoxon rank sum test, and an asterisk is indicated for statistically different means (<0.05) between groups. Scale bars: 1 cm.

Recently, our group developed a class of “AND-Gate” quenched contrast agents that require the multiplexed processing by multiple tumor specific proteases in order to generate a fluorescence signal (Figure S4a).^26,37^ Death-Cat-2, the best performing of this class of probes, is selectively cleaved by both the tumor-associated executioner caspase-3 and the lysosomal cysteine cathepsins. This probe contains two protease substrates that each position a QSY-21 molecule in proximity to a central Cy5 dye (Figure S4b). Optical activation is achieved only when the processing of both substrates occurs, therefore reducing nonspecific probe activation.^26^ However, Death-Cat-2-based tumor detection can still be impacted by nonspecific tissue signals.^26^ To enable logic-gated ratiometric imaging, we synthesized a small-molecule probe, Death-Cat-RATIO, featuring a three-fluorophore FRET system composed of two sulfo-Cy7 dyes and a sulfo-Cy5 dye (Figure 4a and Supplementary Methods). In the 4T1 mouse model of breast cancer, both Death-Cat-2 and Death-Cat-RATIO localized within breast tumors (Figure 4b and c, areas 1 and 2). Similar to 6QC-RATIO, Death-Cat-RATIO equally highlighted the proximal and distal tumors (Figure 4c, panels iv and viii, area 2), while the distal tumors could not be seen by the Cy5 signals only (Figure 4b and c, panels ii, iv, and vii area 2). Next, we assessed tumor detection performance of the probe by carrying out comparative time-dependent imaging of mice with 4T1 breast tumors after injection of Death-Cat-2 and Death-Cat-RATIO (10 nmol; Figure 4d). By 2 h post injection, the TBR of Death-Cat-RATIO (TBR: 4.13 ± 2.66) was significantly higher than DMSO (TBR: 1.06 ± 0.2). It was also elevated, although nonsignificantly, compared to the Death-Cat-2 probe (TBR: 2.15 ± 0.44) at 2 h but by 4 h, was significantly higher (TBR: 5.16 ± 2.59) than the TBR of Death-Cat-2 (TBR: 1.80 ± 0.48) and DMSO (TBR: 1.04 ± 0.2). Twenty-four hours post injection, the TBR of tumors containing Death-Cat-RATIO reached an average value of 7.23 ± 3.72, which is higher than any other AND-gate probe reported.^26^

Contrast agents, including Death-Cat-2, often generate nonspecific signals in organs with high enzymatic activity, such as the liver, or secretory organs where the molecule accumulates, such as kidneys.^26,38^ Therefore, we performed *ex vivo* ratiometric imaging of six types of tissue excised from mice 24 h after probe injection (Figure 4e). For both Death-Cat-2 and Death-Cat-RATIO, we observed significant Cy5 fluorescence signal in kidneys and livers (Figure 4e, Cy5 panels). However, the use of the RATIO channel of Death-Cat-RATIO allowed detection of signal only in true positive tumor samples (Figure 4e, RATIO panel). In mice injected with Death-Cat-2, we quantified an almost identical Cy5 fluorescence signal in liver (signal to tumor: 0.82 ± 0.53) and kidneys (signal to tumor: 1.12 ± 0.3) compared to tumor fluorescence (Figure 4f, left panel). Ratiometric imaging using Death-Cat-RATIO generated a substantial increase in signal selectivity with the ratio signal exclusively detected within the tumor tissue (liver signal to tumor: 0.3 ± 0.04; kidneys signal to tumor: 0.35 ± 0.01; Figure 4f, right panel).

Additionally, metabolites such as reactive nitrogen and oxygen species are known to react with cyanine dyes leading to instability *in vivo*.^39^ Cyanine dyes with longer polymethine chains, such as Cy7, are more prone to oxidative cleavage then dyes with shorter polymethine chains, such as Cy5, resulting in loss of optical properties.^39^ To assess the stability of our probes, we incubated both 6QC-RATIO and Death-Cat-RATIO with physiologically relevant concentrations of peroxynitrite (ONOO^–^) and measured the absorbance of Cy5 and Cy7 to assess loss of signal due to degradation (Figure S5).^40−42^ As expected, peroxynitrite treatment causes a reduction in Cy5 and Cy7 fluorescence indicating degradation of the dye. The Cy7 dye was more sensitive to peroxynitrite than Cy5, but this difference was minimal. However, we only observed modest signal loss (less than 50%) even at the highest physiologically relevant concentration of ONOO^–^ tested (200 nM). These results suggest that even though there is the potential for degradation of the cyanine dyes *in vivo*, our probes retain sufficient stability for use in imaging applications.

### Ratiometric Imaging Enables Selective Detection of Small Tumor Lesions with High Sensitivity and Selectivity

**Figure 5:**
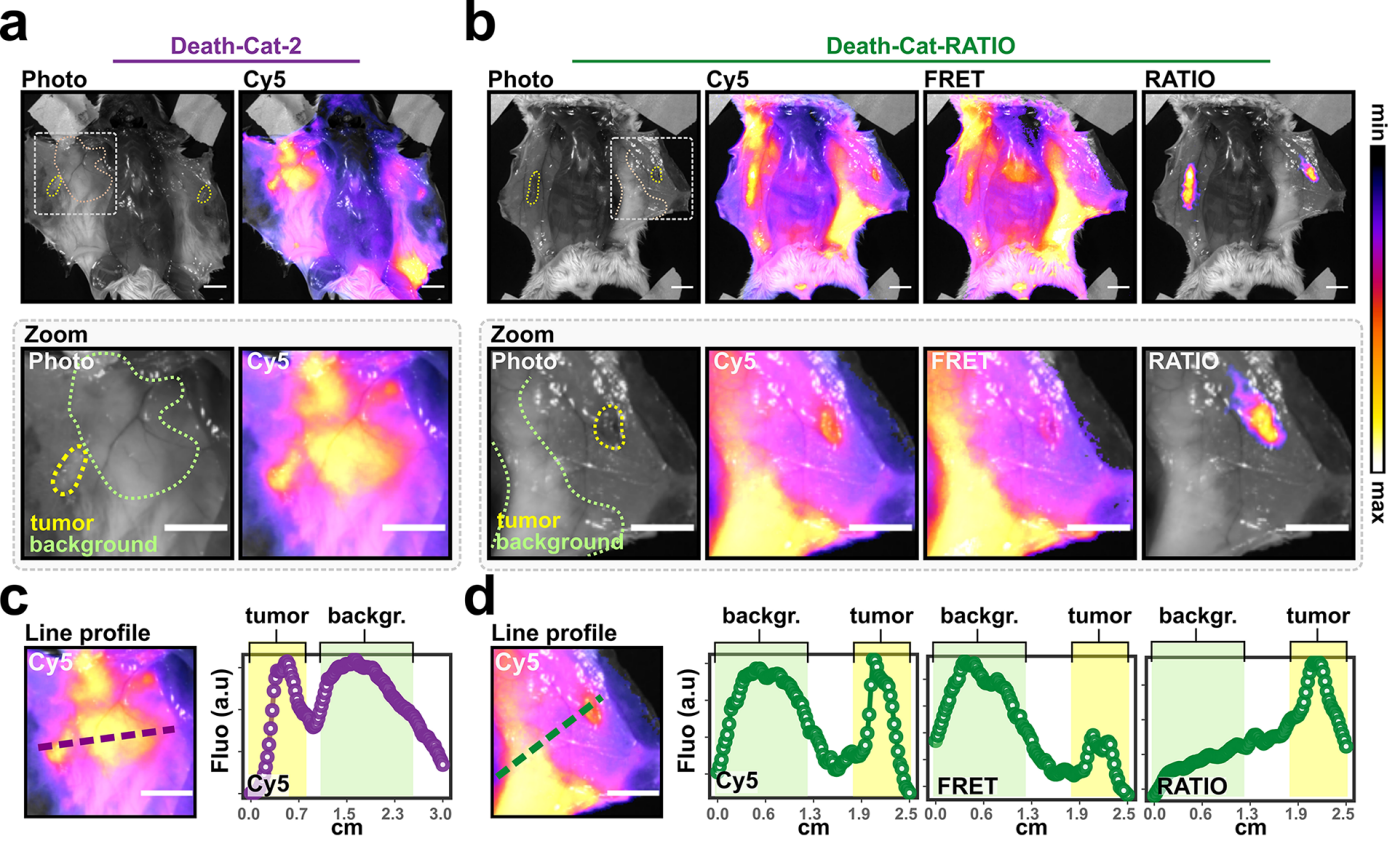
Ratiometric imaging of metastasis-like lesions. (a) Representative fluorescence images showing a splayed mouse with two breast tumors after injection with Death-Cat-2 [10 nmol] 24 h prior to imaging. The left panel shows the brightfield image; the right panel shows the overlay between the Cy5 channel and the brightfield image. The zoom inlet shows a magnification of the tumor. Dashed lines indicate the tumor boundaries and areas of background tissue. (b) Representative fluorescence images showing a splayed mouse with two breast tumors after injection with Death-Cat-RATIO [10 nmol] 24 h prior to imaging. From left to right, the panels show the brightfield image, the overlay between the Cy5 channel and the brightfield image, the overlay between the FRET channel and the brightfield image, and the overlay between the RATIO channel and the brightfield image. The zoom inlet shows a magnification of the tumor. Dashed lines indicate the tumor boundaries and areas of background tissue. (c) Line profile analysis of Death-Cat-2 Cy5 signal. The magnified area (left) shows the line used to generate the data analyzed in the right panel. (d) Line profile analysis of Death-Cat-RATIO. The magnified area (left) shows the line used to generate the data shown in the right panels. Scale bars: 1 cm.

To demonstrate that ratiometric probes are sensitive enough to detect small metastatic cancer lesions, we generated and employed a mouse model in which tumors are roughly the size of the smallest of human metastases. To achieve this, we reduced the number of injected 4T1 tumor cells from 50,000 to 5,000. Upon Death-Cat-2 and Death-Cat-RATIO injection, we could not detect tumor-localized fluorescence signal without splaying of the skin (Figure S6a). The resulting tumors were on average <4.6 mm (some as small as 3.1 mm) in diameter and could not be detected via direct visualization or palpation until skin splaying (Figure S6b). Twenty-four hours post injection, the Cy5 fluorescence from Death-Cat-2 was detected at comparable levels in the lesion and in the surrounding tissue (mean tumor signal: 1.2 × 10^8^ ± 2.0 × 10^7^, mean background signal: 1.2 × 10^8^ ± 2.3 × 10^7^; Figure 5a). We observed a similar pattern for the Cy5 fluorescence for Death-Cat-RATIO (mean tumor signal: 1.0 × 10^8^ ± 1.1 × 10^7^, mean background signal: 1.1 × 10^8^ ± 4.0 × 10^7^; Figure 5b, Cy5 panel). However, the RATIO signal rose significantly above the background at the 24 h time point (Figure 5b, RATIO panel). To further quantify the improvement of Death-Cat-RATIO over Death-Cat-2, we plotted the fluorescence signal along the lines shown in Figure 5c and d. Death-Cat-2 fluorescence was elevated in the tumor and its surroundings (Figure 5c). A similar pattern was observed for the Cy5 signal of Death-Cat-RATIO (Figure 5d, Cy5 plot). Because probe processing occurs exclusively in the tumor, the FRET fluorescence was mainly localized in the background and almost completely absent in the tumor area (Figure 5d, FRET plot). Therefore, the resulting RATIO channel featured a reduced background signal while producing a true positive signal in the small lesion (Figure 5d, RATIO plot). We then calculated a statistically significant difference in the TBR for six metastases-like lesions in mice injected with either Death-Cat-2 (TBR: 1.02 ± 0.2) or Death-Cat-RATIO (TBR: 1.44 ± 0.34; Figure S7).

### Development of a Custom Translatable Device for Ratiometric Imaging-Guided Robotic Surgery of Solid Tumors

**Figure 6:**
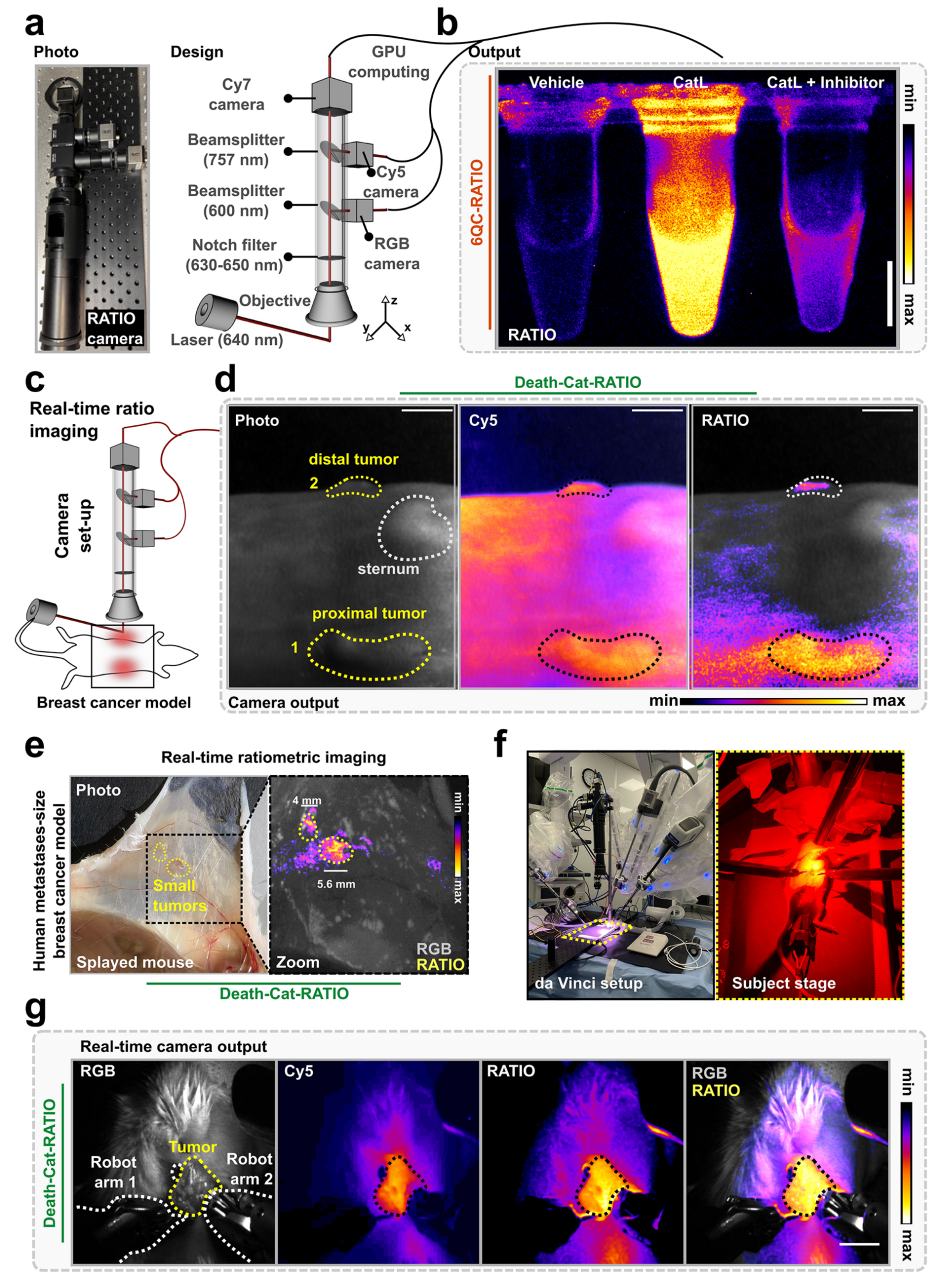
Development and preclinical testing of the ratiometric camera system. (a) Photo and schematic of the components of the camera. (b) Representative images acquired with the camera system showing RATIO channel of vials containing 6QC-RATIO [100 nM] which has been incubated for 30 min with either vehicle, cathepsin L [20 nM], or cathepsin L [20 nM] preincubated 30 min with cathepsin inhibitor E64d [10 μM]. (c) schematic of experimental setup for ratiometric imaging. (d) Representative RATIO camera images showing a mouse carrying two breast tumors injected with Death-Cat-RATIO [10 nmol] 24 h prior to imaging. From left to right, the panels show the brightfield image, the overlay between the Cy5 channel and the brightfield image, and the overlay between the RATIO channel and the brightfield image. The tumor closer to the camera (area 1) is located in the third mammary fat pad; the tumor further from the camera (area 2) is located in the eighth mammary fat pad. Scale bars: 1 cm. (e) Representative RATIO camera images of a mouse model of small 4T1 lesions. Representative white light photo of a splayed mouse (left) and overlay of the RGB signal and RATIO signal acquired with the ratiometric camera (right). (f) Photo of the da Vinci setup for ratiometric robotic surgery with view of the subject stage. (g) Representative images of ratiometric robotic surgery carried out in mice injected with Death-Cat-RATIO [10 nmol] 24 h prior to imaging. Images were acquired with the custom camera system and show, from left to right, the RGB channel, the Cy5 channel, the RATIO channel, and an overlay between the RGB and RATIO channel. Scale bars: 1 cm.

Although imaging device prototypes for ratiometric intraoperative use have been reported, such systems are either not available to the academic community,^43,44^ not designed for NIR imaging,^45,46^ or not suitable for FRET measurements in the NIR region.^10^ Currently no commercial imaging device exists that can be used for real-time simultaneous recording of Cy5 and Cy7 acceptor emission (FRET channel) at video frame rates that are necessary for use during clinical procedures. Therefore, we assembled and tested a custom imaging system that is composed of a 640 nm laser, wide angle diffuser for sample excitation, and three cameras to collect images of white light and the two fluorophores. We used a notch filter that actively excludes incoming laser light to reduce overall background and a dichroic filter that reflects the light with a wavelength below 600 nm to the first RGB camera. We then placed a second camera to collect the light between 600 and 757 nm (Cy5 camera). Finally, we added a third camera to enable Cy7 detection (>757 nm) (Figure 6a, Figure S8, and Table S1). We set up the system to be operated by standard MATLAB code and employed a GPU to compute the real time ratio between the light collected with the two dedicated cameras. In addition to standard camera functions such as gain and laser power adjustments, we implemented several software features to improve the output of the live ratiometric signal. The ratiometric function was designed to compute the ratio between the Cy5 and Cy7 cameras in real time analogous to the post analysis used for the prior experiments with commercial imaging systems. In addition, to avoid signal artifacts when using nonratiometric probes, we implemented a threshold function that excluded background light in the sensitized acceptor channel prior to ratio calculation. We also included an offset feature that, similarly to a camera gain, permitted us to improve signal within field of views characterized by low ratios. As a proof of concept, we used the system to generate ratiometric images of vials containing 6QC-RATIO (Figure 6b). We observed an increased ratio only in vials incubated with the probe and catalytically active cathepsin L. Importantly we detected no or negligible ratios when the probe was mock-treated or incubated with cathepsin L that had been inhibited by E64d (Figure 6b).

To test the feasibility of using the real-time ratiometric system to visualize tumors *in vivo* at high video frame rates, we used the 4T1 mouse model. Mice were injected with Death-Cat-RATIO and imaged 24 h later (Figure 6c). The Cy5 channel showed probe localization in the two breast tumors (Figure 6d, photo and Cy5 panels, areas 1 and 2). However, the Cy5 signal was significantly elevated in background areas and showed low contrast between the distal tumor signal and the adjacent healthy tissue (Figure 6d, Cy5 panel, area 2). In contrast, the real-time RATIO channel produced an improved tumor to background signal and a pronounced contrast between the distal tumor and its surroundings (Figure 6d, RATIO panel, area 2). We then investigated whether the camera could detect small lesions which are comparable in size to human metastasis. To do so, we generated mice carrying cancer lesions which were not identifiable via skin observation or palpation, injected them with Death-Cat-RATIO, and splayed the skin containing the tumors before imaging with the ratiometric camera (Figure 6e, left). Real time imaging showed RATIO signal above background, correctly colocalizing with two lesions of 5.6 and 4 mm in diameter, respectively (Figure 6e, right). Finally, to improve the performance of ratiometric probes and camera for robotic surgery, we coupled the ratiometric device to the FDA-approved da Vinci Xi Surgical System (Figure 6f). After injection, the Death-Cat–RATIO probe produced fluorescence contrast in the tumor, clearly highlighting the margins in real time during surgery and enabling successful robotic resection (Figure 6g and Supplementary Movies 1 and 2).

## Discussion

A primary goal of molecular imaging of cancer is to develop contrast agents that enable real-time, accurate, and specific detection of tumor margins. However, single parameter detection remains prone to artifacts even in the NIR region, since interference from a variety of analyte-independent factors can negatively impact imaging outcome. In fact, in clinical trials evaluating the intraoperative use of the NIR-dye ICG for ovarian and liver cancer treatment, 25% to 62% of the resected samples were false positives.^47^

We hypothesized that ratiometric imaging could reduce the number of false positives and negatives as well as improve the detection of difficult to detect small lesions. Therefore, we developed and tested the ratiometric protease activated probes 6QC-RATIO and Death-Cat-RATIO *in vitro* and *in vivo*. In a mouse model of breast cancer, the probes reached TBR values at 24 h post-injection that, to our knowledge, are among the highest of published protease activated fluorescent NIR contrast agents (Figures 3c and 4d).^7^

Optical surgical navigation is generally influenced by camera positioning and distance from the area of interest, which, when suboptimal, prevents the identification of out-of-focus tumor lesions. In this work, we demonstrate that ratiometric imaging of 6QC-RATIO and Death-Cat-RATIO successfully highlight distal tumors that could only be detected by single parameter detection upon correct positioning of the camera system (Figures 3b and 4c). In addition, we demonstrated that ratiometric probes significantly reduced nonspecific signal in organs heavily affected by probe accumulation due to metabolism (Figure 4e). This is because accumulation of ratiometric probes within organs with low enzyme activity will not increase the RATIO channel as the intact probe will produce FRET signal. In addition, the ability of the probes to correctly identify false positives could reduce the removal of excessive tissue leading to damage and aesthetic defects during tumor surgery. By employing a ratiometric detection modality, we could correctly assign true positives to small lesions resembling, in size, human metastasis which were otherwise undetected due to poor TBR in single parameter detection (Figure 5). Therefore, ratiometric measurements have the potential to improve surgical margin detection in small metastasis in high-risk patients.

Using commercially available *in vivo* imaging systems, we were only able to compute and process ratiometric images after the acquisition of the data (Figures 2, 3, 4, and 5). Therefore, we developed a custom camera system and imaging device that produced ratiometric movies of the target tissue with a frame rate suitable for intraoperative imaging applications. We evaluated the imaging device in combination with the FDA-approved da Vinci Xi robot and were able to detect and resect breast tumors via robotic ratiometric image-guided surgery (Figure 6).

Our imaging approach is designed to be compatible with NIR spectroscopy applications. However, recently, polymethine dyes that enable multicolor imaging in the shortwave-infrared region (SWIR, 1000–2000 nm) have been developed.^9,48^ Therefore, it is likely that the use of SWIR dyes as FRET pairs in ratiometric probes will further improve imaging depth and tumor-to-background signals.^48,49^ In future work, the ratiometric imaging device presented here could be converted into an endoscope compatible with laparoscopic and endoscopic procedures by using the same design principles and software. For example, elevated background signals in the colon generally hamper sensitive and accurate fluorescence-guided applications and white light endoscopy remains the gold standard in colorectal cancer diagnosis.^50,51^ Therefore, we envision that endoscopic surveillance of colorectal cancer will be greatly benefited by the use of ratiometric probes and a compatible NIR or SWIR imaging device. Ultimately, we believe that 6QC-RATIO and Death-Cat-RATIO mediated detection of clinically significant lesions using ratiometric real time imaging will positively impact surgery outcomes while also potentially being useful for early diagnostic prevention of cancers such as colorectal cancer.

## Supporting Information Available

Supplementary figures, supplementary tables, synthetic schemes, biology methods, chemistry methods, HPLC purity analysis, animal experiment statement, risk statement, and references for protocols (PDF)
Movie 1: Ratiometric interoperative imagining setup (MP4)
Movie 2: Interoperative imaging (MP4)
Transparent Peer Review report available (PDF)

## Supplementary Material

oc3c00261_si_001.pdf

oc3c00261_si_002.mov
