# Supplementary material for: Protease Activated Probes for Real-Time Ratiometric Imaging of Solid Tumors
Source: ACS Cent Sci. 2023 May 4;9(5):1059–69. doi: 10.1021/acscentsci.3c00261 (PMC10214504; doi:10.1021/acscentsci.3c00261)
Supplement: Supplementary file 1 — oc3c00261_si_001.pdf [file oc3c00261_si_001.pdf]

**Protease activated probes for real-time ratiometric imaging of solid tumors**

Franco F. Faucher[a], Kevin J. Liu[b], Emily D. Cosco[c], John C. Widen[c,d], Jonathan Sorger[e], Matteo Guerra[c,f,*] and Matthew Bogyo[c, g, h,*]

[a]Department of Chemistry, Stanford University, Stanford, CA, 94305, USA;

[b]Program in Cancer Biology, Stanford University School of Medicine, Stanford, CA, 94305 USA;

[c]Department of Pathology, Stanford University School of Medicine, Stanford, CA 94305, USA;

[d]Present address: Denali Therapeutics Inc., 161 Oyster Point Blvd., South San Francisco, CA 94080, USA;

[e]Intuitive Surgical Inc., Sunnyvale, CA, 94086, USA;

[f]Present address: Department of Biochemical and Cellular Pharmacology, Genentech, 1 DNA Way, South San Francisco, 94080, USA;

[g]Department of Chemical and Systems Biology, Stanford University School of Medicine, Stanford, CA 94305, USA;

[h]Department of Microbiology and Immunology, Stanford University School of Medicine, Stanford, CA, 94305, USA.

[*]To whom correspondence may be addressed. Email: guerrm22@gene.com or mbogyo@stanford.edu

**Table of Contents**

Supplementary Figures ....................................................................................................3
Schemes ....................................................................................................12
Methods....................................................................................................14
Biology Methods....................................................................................................14
Chemistry Methods ....................................................................................................16
HPLC Purity Analysis ....................................................................................................22
Animal Experiment Statement....................................................................................................24
Risk Statement ....................................................................................................24
References ....................................................................................................24

## Supplementary Figures

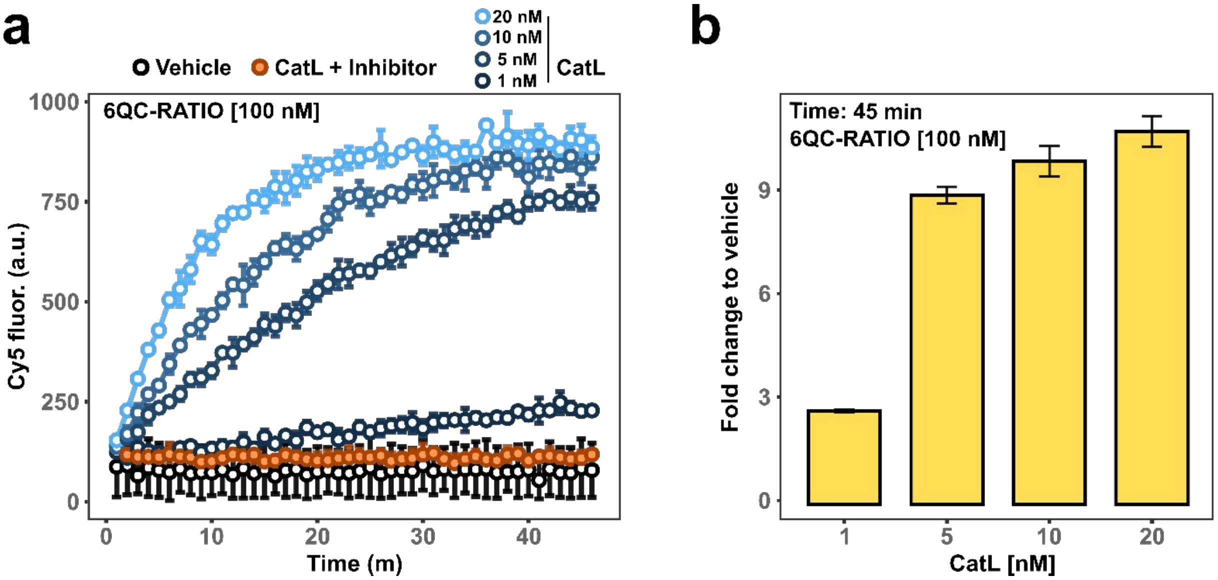


**Figure S1**. In vitro characterization of FRET performance upon cathepsin L cleavage. A) Fluorimetric time course analysis of 6QC-RATIO cleavage upon cathepsin L addition (at 1, 5, 10 or 20 nM concentration), vehicle addition or addition of cathepsin L [10 nM] preincubated 30 min with a cathepsin inhibitor E64d [10 µM]. B) Bar graph showing the fold change in Cy5 fluorescence emission 45 min after addition of cathepsin L showing dequenching of Cy5 upon cathepsin processing. Data are shown as mean ± sd of three technical replicates.

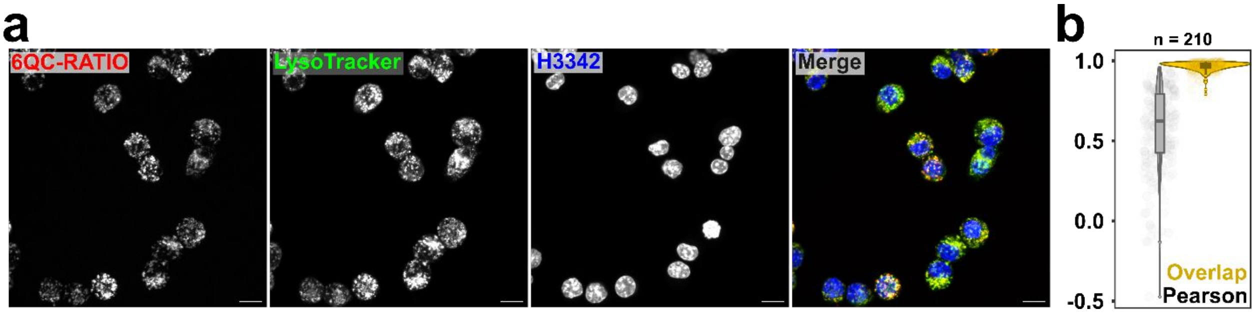


**Figure S2.** A) Representative maximal projection of confocal microscopy stacks of RAW 264.7 cells incubated with 6QC-RATIO [1µM] for 2 hours and LysoTracker [3.2 nM] and H3342 [16 µM] for 30 min prior to imaging. From left to right, 6QC-RATIO, LysoTracker, Hoechst 3342 and merge channels are shown. Scale bars: 10 µm. B) Violin, box and point plots showing colocalization analysis (Pearson correlation coefficient 0.57±0.26; Area overlap0.96±0.02, n = 210) between 6QC-RATIO and LysoTracker signal for 210 macrophage granules.

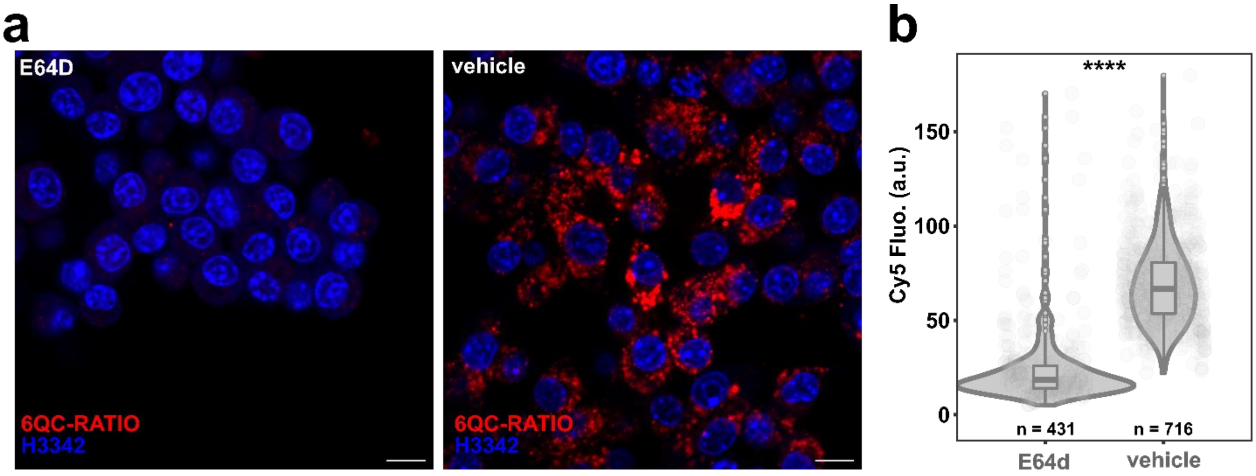


**Figure S3.** A) Left, representative maximal projection of confocal microscopy stacks of RAW 264.7 cells treated with E64d [100 μM] for 1.5 hours and incubated with 6QC-RATIO [1 μM] for 2 hours] and H3342 [16 μM] for 30 min prior to imaging. Right, representative maximal projection of confocal microscopy stacks of RAW 264.7 cells treated with vehicle (DMSO) for 1.5 hours and incubated with 6QC-RATIO ([1 μM] for 2 hours) and H3342 [16 μM] for 30 min prior to imaging. Scale bars: 10 μm. B) Violin, box and point plots showing Cy5 signal in cells treated with E64d (24.7 ± 21.8, n = 431) or vehicle (69.3 ± 22.1, n = 716). Statistics were calculated via Wilcoxon rank sum test.

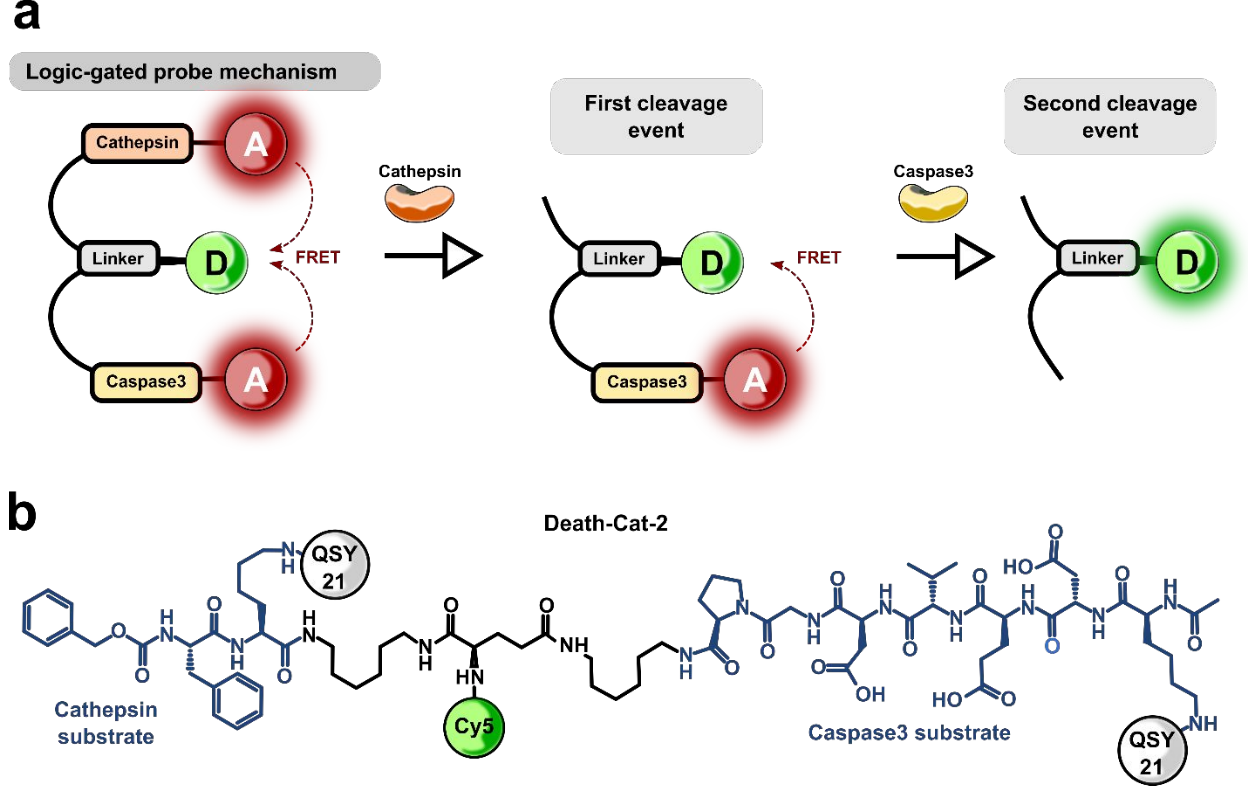


**Figure S4.** A) Schematic of mechanism of logic-gated 'AND-Gate' quenched contrast agents that require the multiplexed processing by cathepsins and caspase 3 proteases to generate a fluorescence signal in the tumor. B) Chemical structure of Death-Cat-2.

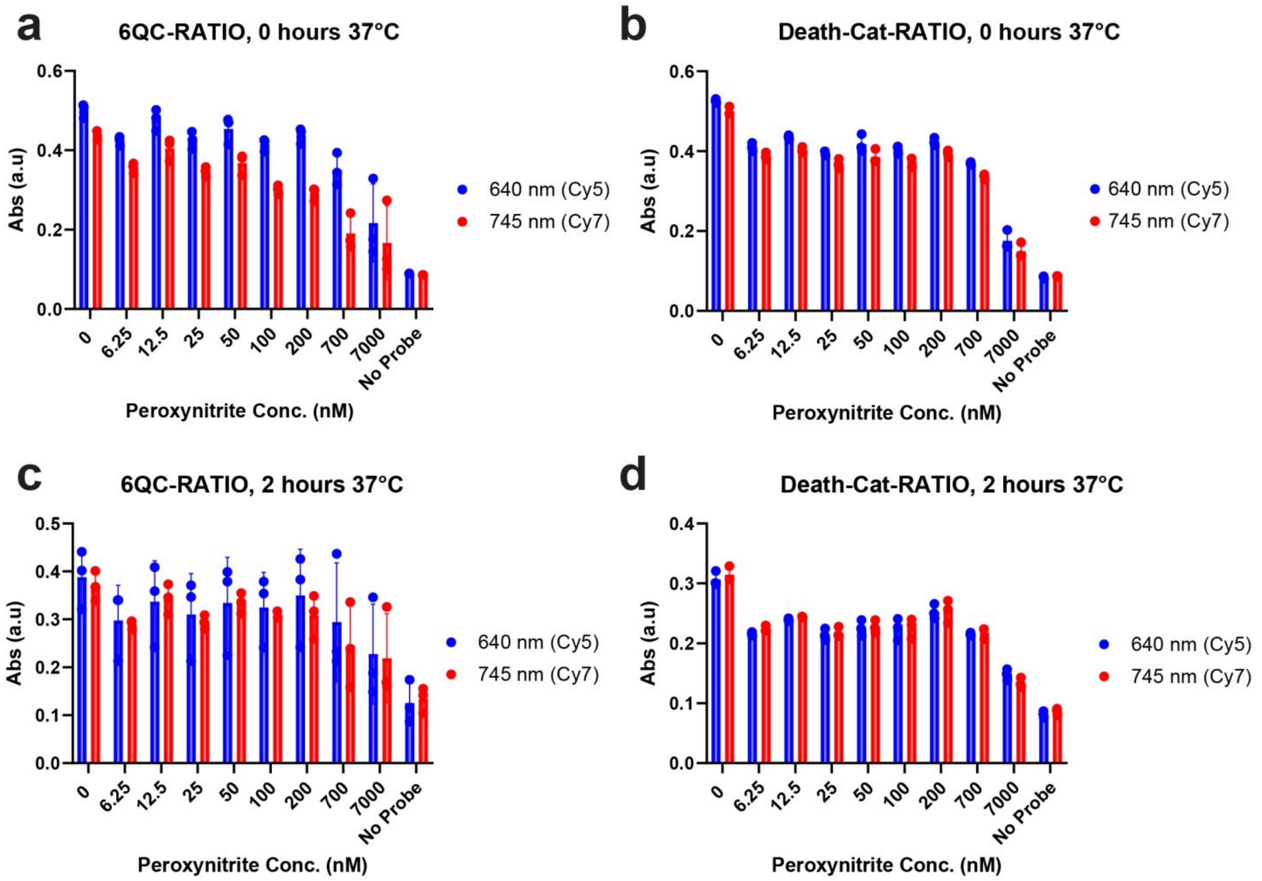


**Figure S5.** Degradation assay of cyanine dyes with freshly generated peroxynitrite a reactive nitrogen oxide species. 6QC-RATIO or Death-Cat-RATIO at 7uM (physiological concentration) were incubated with peroxynitrite and absorbance was measured at both 640 nm and 745 nm. Time points were taken immediately after addition and 2 hours after incubating at 37° C. A) Absorbance for 6QC-RATIO at 0 hours. B) Absorbance for Death-Cat-RATIO at 0 hours. C) Absorbance for 6QC-RATIO at 2 hours. D) Absorbance for Death-Cat-RATIO at 2 hours. Data are shown as mean ± sd of three technical replicates.

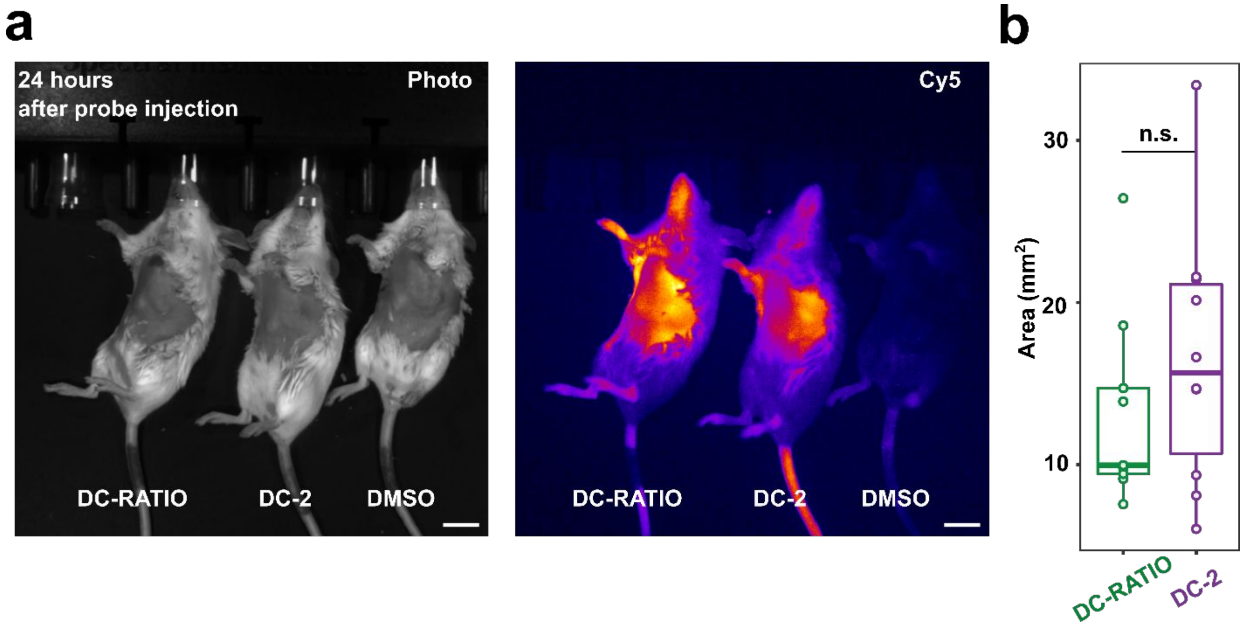


**Figure S6.** A) Representative images of mice injected with either Death-Cat-RATIO (DC-RATIO), Death-Cat-2 (DC-2) or DMSO. Mice were injected 5 days prior imaging with 5000 4T1 breast tumor cells. Scale bars: 1 cm. B) Boxplot showing tumor area quantification after mouse skin splaying (DC-RATIO: 13.2±6.04 $mm^2$; DC-2: 16.6±8.08). Statistics were calculated via Wilcoxon rank sum test.

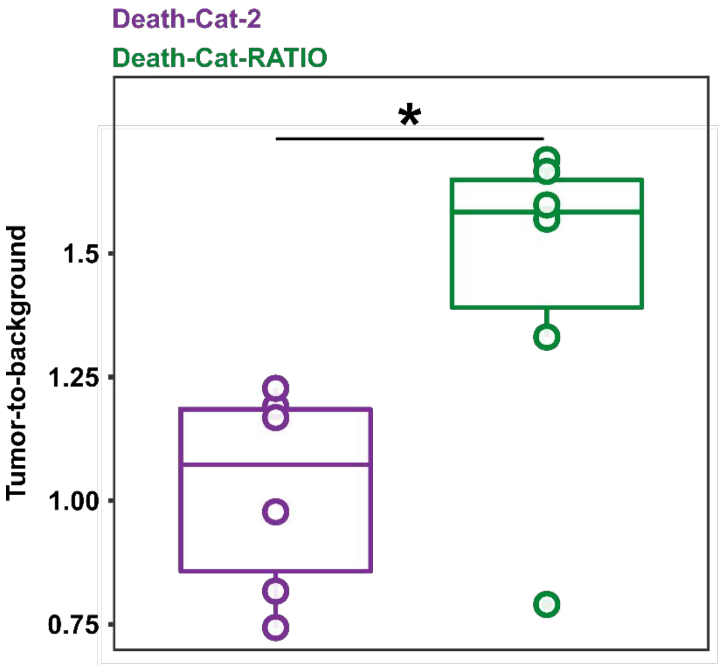


**Figure S7.** Box and point plot showing the tumor to background signal calculated from six splayed small tumors in the 4T1 mouse model. Statistics were calculated via Wilcoxon rank sum test.

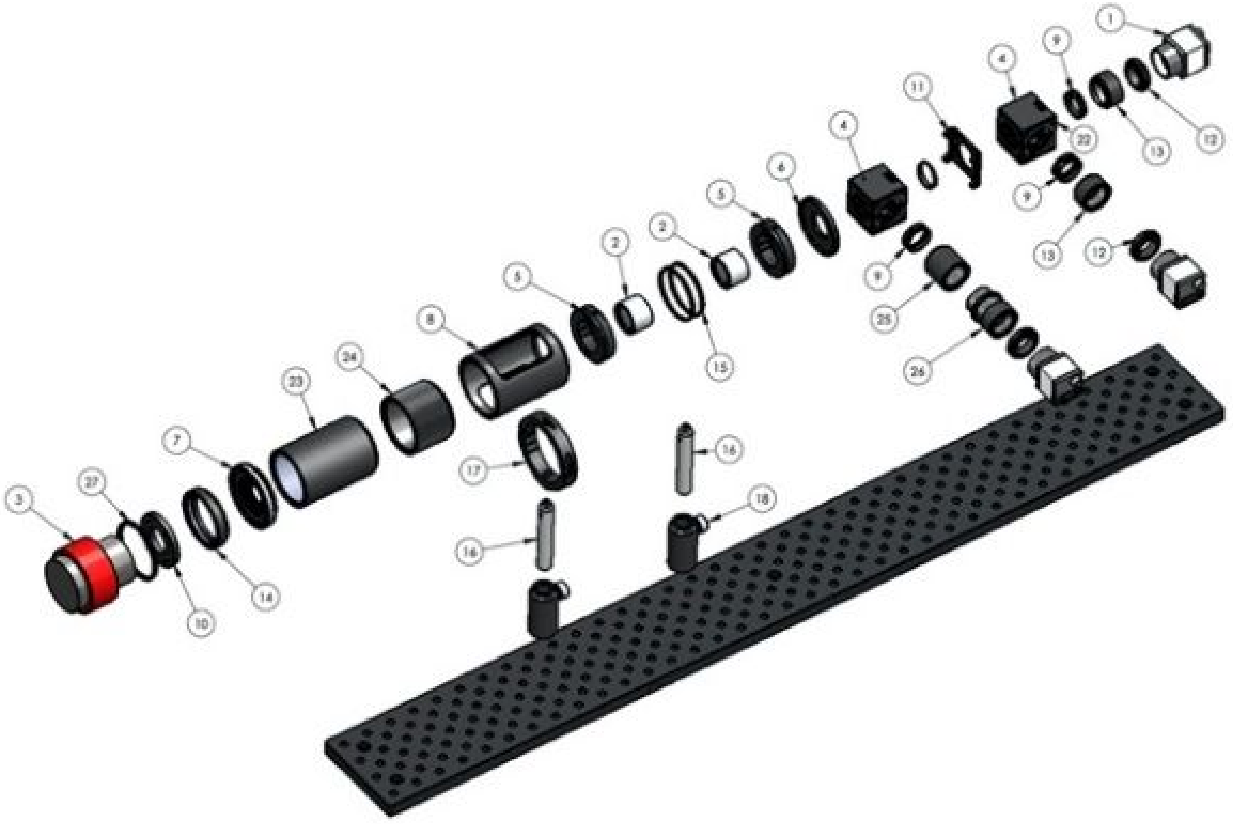

**Figure S8.** The design of the imaging system. To allow for maximum flexibility, a three-camera setup was designed and built to allow imaging of Cy5, Cy7 and white light via the placement of standard dichroic filters. 2/3”, 5 Megapixel CMOS sensors with an acquisition rate of 78 frames per second to approximate data rates and image quality from a clinical system. A 642 ± 25 nm notch filter is placed behind the lens in order to block out the 650 nm laser source used for excitation of the Cy5 fluorophore. Cy5 emission was captured by one camera. Since the 650 nm laser falls significantly below the excitation curve for Cy7, the only excitation of the Cy7 fluorophore derived from Cy5 emission, whose tail diminishes around 750 nm. A long-pass filter was placed in front of the second camera that will capture Cy7 emission photons. A third camera to produce white light images was also available for positioning and observation. See separate Supplementary Materials spreadsheet for details on camera parts.

**Table S1**

List of parts used to build the camera system shown in Supplementary Fig 6.

| PART NUMBER | Vendor | Correspondence to scheme in Supplementary Figure 6 (when applicable) |
|---|---|---|
| SM2NT | ThorLabs | 27: SM2NT - SM2 (2.035"-40) Locking Ring, 2.25" Outer Diameter |
| SM1V10 | ThorLabs | 26: SM1V10 - Ø1" Adjustable Lens Tube, 0.81" Travel Range |
| SM1L10 | ThorLabs | 25: SM1L10 - SM1 Lens Tube, 1.00" Thread Depth, One Retaining Ring Included |
| SM2L15 | ThorLabs | 24: SM2L15 - SM2 Lens Tube, 1.5" Thread Depth, One Retaining Ring Included |
| SM2L30 | ThorLabs | 23: SM2L30 - SM2 Lens Tube, 3" Thread Depth, One Retaining Ring Included |
| 97763A318 | McMaster | Stainless Steel Button Head Hex Drive Screws |
| SM1CP2 | ThorLabs | SM1CP2 - Externally SM1-Threaded End Cap |
| SM1RR | ThorLabs | SM1RR - SM1 Retaining Ring for Ø1" Lens Tubes and Mounts |
| MB436 | ThorLabs | MB436 - 4" x 36" x 1/2" Aluminum Breadboard, 1/4"-20 Double-Density Taps |
| PH2 | ThorLabs | 18: PH2 - Ø1/2" Post Holder, Spring-Loaded Hex-Locking Thumbscrew, L = 2" |
| SM2TC | ThorLabs | 17: SM2TC - Clamp for SM2 Lens Tubes |
| TR3 | ThorLabs | 16: TR3 - Ø1/2" Optical Post, SS, 8-32 Setscrew, 1/4"-20 Tap, L = 3" |
| SM2RR | ThorLabs | 15: SM2RR - SM2 Retaining Ring for Ø2" Lens Tubes and Mounts |
| SM2L03 | ThorLabs | 14: SM2L03 - SM2 Lens Tube, 0.3" Thread Depth, One Retaining Ring Included |
| SM1L05 | ThorLabs | 13: SM1L05 - SM1 Lens Tube, 0.50" Thread Depth, One Retaining Ring Included |
| SM1A39 | ThorLabs | 12: SM1A39 - Adapter with External C-Mount Threads and External SM1 Threads, 3.2 mm Spacer |
| CM1-CC | ThorLabs | 11: CM1-CC - Cage Cube Connector for Compact 30 mm Cage Cubes |
| SM2A54 | ThorLabs | 10: SM2A54 - Adapter with External SM2 Threads and Internal C-Mount Threads, 5.1 mm Long |
| SM1W741 | ThorLabs | SM1W741 - Wedge Prism Mounting Shim, 7° 41' Wedge Angle |
| SM2L30C | ThorLabs | 8: SM2L30C - SM2 Slotted Lens Tube, 3" Thread Depth, 2 Retaining Rings Included |
| SM2D25D | ThorLabs | 7: SM2D25D - SM2 Ring-Actuated Iris Diaphragm (Ø1 - Ø25 mm) |
| SM1A2 | ThorLabs | 6: SM1A2 - Adapter with External SM1 Threads and Internal SM2 Threads |
| SM2A21 | ThorLabs | 5: SM2A21 - Externally SM2-Threaded Mounting Adapter with Ø1.20" (Ø30.5 mm) Bore and 2" Outer Diameter |

| | | |
|---|---|---|
| CM1-DCH | ThorLabs | 4: CM1-DCH - 30 mm Cage Cube with Dichroic Filter Mount |
| Lens | | |
| 135mm EFL Triplet #64-839 | Edmund Optics | 2: 135mm EFL $MgF_2$ Coated, UV-NIR Corrected Triplet |
| IDS Camera U3-3080CP (1 color, 2 mono) | IDS | |

## Schemes

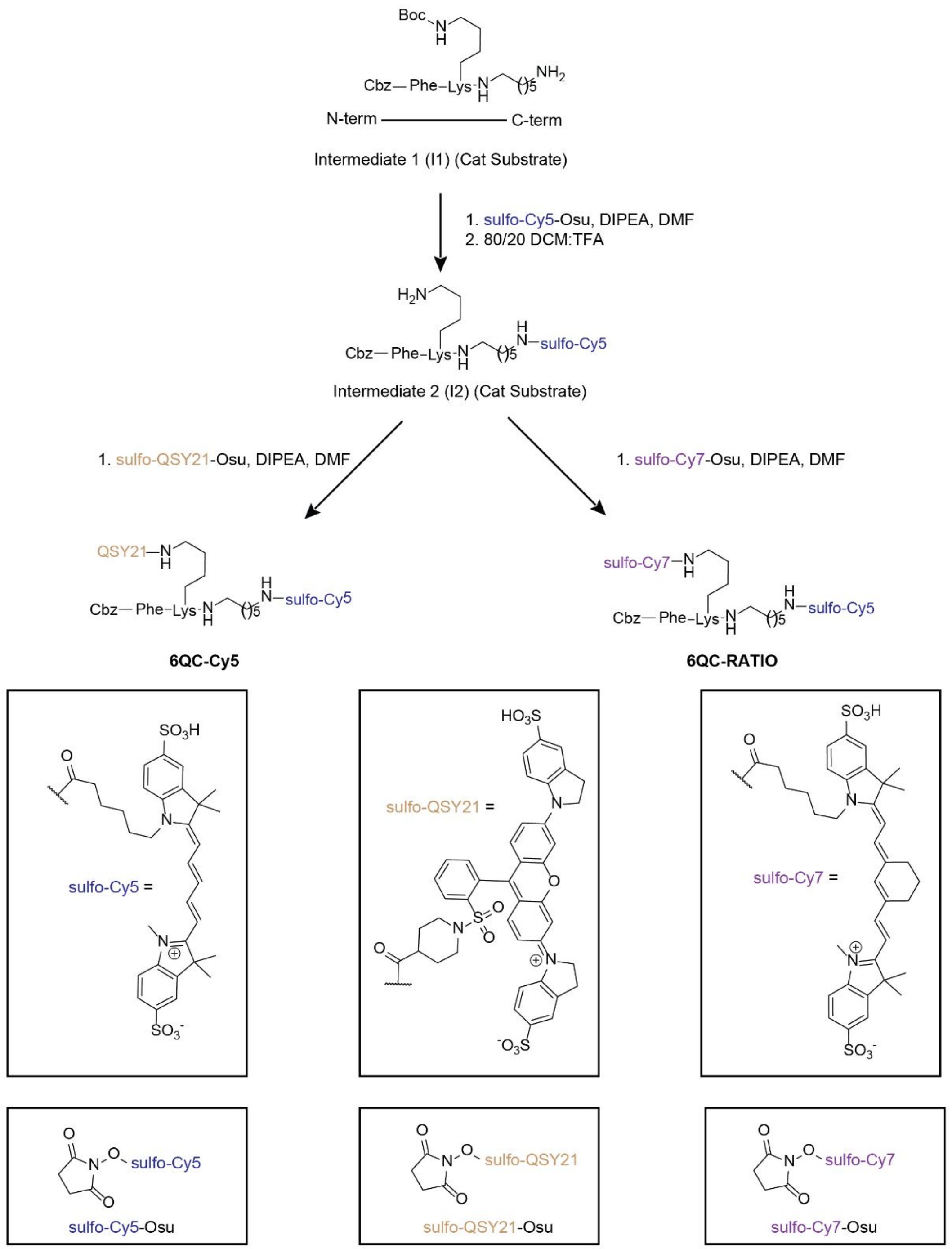


**Scheme S1.** Synthesis of **6QC-Cy5** and **6QC-RATIO**.
See methods section for synthetic details.

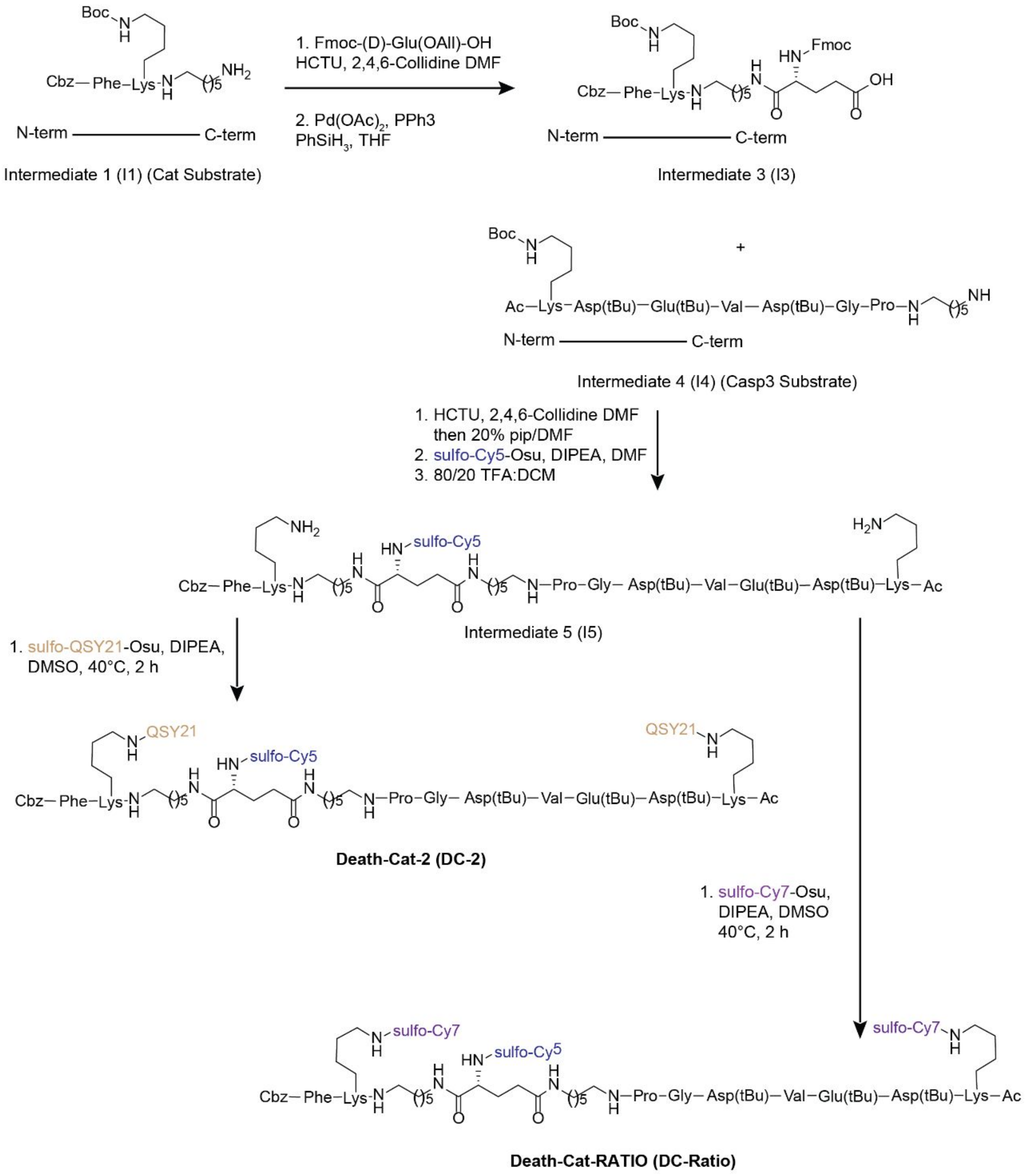


**Scheme S2** Synthesis of **Death-Cat-2** and **Death-Cat-RATIO**
See methods section for synthetic details.

## Methods

## Biology Methods

### General cell culture

4T1 cells (ATCC CRL-2539) were cultured in Roswell Park Memorial Institute (RPMI, Corning, 10-040-CV) 1640 medium containing 2 g l−1 of glucose, 0.3 g ml−1 of L-glutamine, and supplemented with 10% FBS and 100 U ml−1 penicillin and 100 µg ml−1 streptomycin. RAW246.7 (ATCC TIB-71) macrophages were cultured in Dulbecco's modified Eagle's medium (DMEM, Gibco, 11965-092) containing 4.5 g l−1 of glucose supplemented with 10% fetal bovine serum (FBS, GeminiBio, 100602), and 100 U ml−1 penicillin and 100 µg ml−1 streptomycin (Gibco, 15140-122). In general, 4T1 and RAW246.7 were passaged 3 times after thawing before injection or confocal microscopy.

### Fluorogenic Substrate Cleavage Assay

Recombinant cysteine cathepsin L was from R&D Systems (Catalog No.: 952-CY). Buffers used for fluorogenic substrate cleavage assays were made as previously described 24,26,47. The reducing agent 1,4-dithiothreitol was freshly added to buffers immediately before use. All assays were conducted in clear Eppendorf tubes. Compounds [10 mM] were diluted from DMSO stock solutions in the cathepsin L buffer. Immediately before fluorescence measurements, protease was added to the final concentration reported in the text. Fluorescence were performed with a Lago-X instrument (Spectral Instruments Imaging manufacturer, Tucson, AZ, USA). Cy5 signal was detected by exciting at 640 nm and fluorescence emission was recorded at 690 nm. FRET (Cy7 sensitized emission) was recorded by exciting at 640 nm and recorded at 790 nm. Cy7 signal was detected by exciting at 745 nm its emission was recorded at 790 nm. Exposure times were kept to 15 seconds for the Cy5 and FRET channel, and 5 seconds for the Cy7 channel. All experiments were repeated at least two times and a minimum of two technical replicates were recorded. Measurements were carried out for 26 minutes.

### Live-cell fluorescence microscopy assay

To prove the colocalization between 6QC-RATIO and LysoTrackerTM Green (Thermo Fisher, Cat. No.: L7526), 1e5 RAW macrophages were seeded in 8 well-microscopy slides for 24 hours in complete DMEM (w/ 10% FBS,1% Pen/Strep, 300 µL). After washing and replacing medium with fresh DMEM (300 µL), 6QC-RATIO was added to cells (final conc. of 1µM) and incubated for 2 h 15' at 37°C, 5%CO2. After washing, Hoechst 33342 (Invitrogen, Cat. No.: H3570) was added to a final concentration of 16 µM and LysoTrackerTM Green was added to a final concentration of 3.2 nM per well in DMEM without phenol red. Imaging was started 5 min after Hoechst 3342 and LysoTrackerTM addition. To prove that 6QC-RATIO is cathepsin dependent, 1e5 RAW macrophages were seeded in 8 well-microscopy slides for 24 hours in complete DMEM (w/ 10% FBS,1% Pen/Strep, 300 µL). After washing and replacing medium with fresh DMEM (300 µL), either E64d (Santa Cruz Biotechnology, Cat. No.: sc-201280A) or vehicle (DMSO) were added to a final concentration of 100 µM and incubated for 1 h 40' at 37°C, 5 % CO2. After, 6QC-RATIO was added to a final concentration of 1 µM and incubated for 2h at 37°C, 5% CO2. After washing, Hoechst 33342 (Invitrogen, Cat. No.: H3570) was added to a final concentration of 16 µM and LysoTrackerTM Green was added to a final concentration of 3.2 nM per well in DMEM. To avoid

dye diffusion out of lysosomes, after 15’ cells were fixed with paraformaldehyde (4 % in PBS) for 15’ at 37°C. Samples were then washed with 300 μL of PBS, resuspended in 300 μL of PBS and imaged at room temperature. Confocal microscopy images were acquired a ×40 oil-emersion objective on a Zeiss LSM700 confocal microscope. Hoechst 33342 was excited with the 405 UV laser line. LysoTrackerTM Green was excited with the 488 nm laser and its emission sampled recorded after 500 nm. Cy5 direct excitation was carried out with the 639 nm laser and sampled recorded after 645 nm. Pinhole size, laser power and gains were set at the beginning of image acquisition and kept constant throughout the same experiments. Experiments were performed at least twice and technical duplicates were imaged.

**Image analysis**

Confocal images were analyzed by FIJI (V1.51g) and the ImageJ macro “FluoQ” (version 3-97) 48. The macro operates a background subtraction by ImageJ’s built-in rolling ball function, then, it allows the operator to set manually a channel threshold. Finally, images are smoothed via a median filter. Region of interest (ROIs) are selected manually by drawing their border with the pencil tool. When z stacks were processed, the Z-projection of the maximal intensity of the donor channel was used as channel for cell segmentation. The same macro was used for colocalization analysis and Pearson correlation coefficient and Area overlap calculation. For quantitative analysis of fluorescence signal in mice, images and movies were exported as TIF files by using the Aura Software (Spectral Instruments Imaging, Tucson, AZ, USA) and then analyzed via FIJI (V1.51g) and the ImageJ macro “FluoQ” (version 3-97) 48. For analysis, the signal within an ellipsoid region of interest surrounding the tumor was used to calculate the tumor average fluorescence pixel intensity. To generate the RATIO image, the change in donor/acceptor ratio was then calculated as mean pixel intensity of each ROI (over time in case of time series) from the Cy5 and FRET channels and the ratio (RATIO channel) was computed, as the division between the donor (Cy5) and acceptor (FRET) channels. For tumor-to-background calculations, the average signal for each tumor was divided by the average signal of directly adjacent healthy tissue with a similar ellipsoid. For 6QC-Cy5 and Death-Cat-2 probes, the tumor to background was calculated as Cy5 fluorescence intensity in the tumor region divided by the Cy5 fluorescence intensity in the surrounding background region. For 6QC-RATIO and Death-Cat-RATIO probes, the tumor to background was calculated as the Cy5 fluorescence intensity in the tumor subtracted of the Cy5 fluorescence intensity in the background, divided by the FRET fluorescence intensity in the tumor subtracted of the FRET fluorescence intensity in the background.

**4T1 breast tumor model**

One hour before injection, 4T1 cells were trypsinized by addition of pre-warmed trypsin (Gibco, Cat. No.: 25300062) at 37°C for 3 min and detached with pipetting. Two volumes of growth medium were added and the cells were centrifuged at 250 x g for 3 min and the media removed. ,Cells were then washed three times with PBS, resuspended in PBS (100 µl, 1 × 106 cells per ml) or (100 µl, 5 × 104 cells per mL, for metastasis like experiments) and injected subcutaneously into the third and eighth mammary fat pads of BALB/c female mice (aged 6–8 weeks; Jackson Laboratory) while the mice were under isoflurane anesthesia. Mice were injected with probe for imaging between 7–10 days (5 days to generate small lesions) after seeding. Body hair was removed from the tumor site, chest, and surrounding areas using Nair lotion while the mice were

under anesthesia 1 h before imaging. 10 nmol of probe were dissolved in 1× PBS (10% DMSO, 30% PEG 400) and injected i.v. in the tail vein (100 µl) using a 1 ml insulin syringe (28 gauge) while under anesthesia. After injection, mice were imaged noninvasively at the indicated time points using the Lago-X (Spectral Instruments Imaging). For ex vivo organs analysis, mice were euthanized under isoflurane anesthesia by cervical dislocation after in vivo imaging. Tumors and various organs (tumor, fat pad, heart, lung, liver, kidney, spleen) were isolated by dissection and imaged ex vivo to measure the biodistribution profiles of the probes using the using the Lago-X (Spectral Instruments Imaging).

**Degradation Assay of Cyanine Dyes**

Peroxynitrite was generated freshly as previously described in Robinson et. al [1]. Peroxynitrite concentration was determined as previously described in Chen et. al [2]. The physiological concentration of probe was determined using 10 nmols of probe in 1.5 mL of blood. Therefore, 15 uL of 7 uM probe (physiological concentration) was incubated with varying concentrations of peroxynitrite 15 uL (physiological concentration) and activated upon addition of 15 uL of 1M HCl [3]. 6QC-RATIO or Death-Cat-RATIO were measured at both 640 nm and 745 nm. Time points were taken immediately after addition and 2 hours after incubating at 37° C.

**Robotic fluorescence-guided surgery**

Breast tumor-bearing mice (4T1) were administered with the indicated probes in the tail vein 24 h before surgery as described above. Robotic fluorescence-guided surgery was performed using an FDA-approved da Vinci Xi® surgical system in parallel with our custom camera. Surgery on breast-tumor-bearing mice was performed under inhaled isoflurane. For presurgical pain relief, buprenorphine (0.05 mg $kg^{-1}$) was administered intraperitoneal (i.p.) while under inhaled anesthesia. After resection of the tumors, mice were put into deep sedation with ketamine–xylazine solution (80 mg $kg^{-1}$ ketamine, 10 mg $kg^{-1}$ xylazine, i.p.), followed by cervical dislocation. Breast cancers were detected using a combination of white light and fluorescence signal based on probe activation as a guide to determine tumor margins from healthy tissue

**Chemistry Methods**

**Materials and Synthetic Methods**. All reactions were performed exposed to atmospheric air and with solvents not previously dried over molecular sieves or other drying agents, unless specified. Reactions containing light sensitive materials (such as QSY21, sulfo-Cy5, sulfo-Cy7) were protected from light. The ACS reagent grade N,N'- dimethylformamide (DMF), tetrahydrofuran (THF) containing 250 ppm of butylated hydroxy toluene (BHT), molecular biology grade dimethyl sulfoxide (DMSO), and all other commercially available chemicals were used without further purification. All reactions were performed at room temperature unless specified. Reaction progress and purity analysis was monitored using an analytical LC-MS. The LC-MS systems used was either an Agilent 1200 HPLC equipped with an Agilent Zorbax SB-C18 column (1.8 µm, 2.1 x 50 mm) coupled to an Agilent 6125B Single Quad Mass Spectrometer or an Agilent 1100 Series HPLC equipped with a Luna 4251-E0 $C_{18}$ column (3 µm, 4.6 x 150 mm) coupled to a PE SCIEX API 3000 mass spectrometer (wavelengths monitored = 215, 254, 600 nm). High Resolution Mass Spectrometry (HRMS) samples were diluted in 50:50:0.1 water/acetonitrile/formic acid and

immediately analyzed by infusion ESI/MS on the Thermo Exploris 240 BioPharma Orbitrap mass spectrometer. Spectra were collected in full scan MS mode, Orbitrap resolution 120000, in negative ionization mode. Purification of intermediates and final compounds was carried out using a CombiFlash Companion/TS (Teledyne Isco) with a 4 or 12 g reverse phase $C_{18}$ RediSep Rf Gold column (wavelengths monitored = 215 & 254 nm). Information regarding gradient programs for purifications can be found in the Chemistry Protocols section below. Intermediates were identified by their expected m/z using LC-MS.

**Chemistry Protocols.**

Synthetic Methods were adapted from Widen, J. et. al. AND-gate contrast agents for enhanced fluorescence-guided surgery and Ofori, L. et. al. Design of Protease Activated Optical Contrast Agents That Exploit a Latent Lysosomotropic Effect for Use in Fluorescence-Guided Surgery [4,5]. In general, sulfo-Cy7 can replace QSY21 as a reactant for synthesis of fluorogenic probes.

**Solid Phase Peptide Synthesis**. The Cat Substrate (I1) Casp3 substrate (I4) and negative control substrates were synthesized on 2-Chlorotrityl resin using standard Fmoc chemistry as previously described [5]. Peptides were cleaved from resin using 1,1,1,2,2,2-hexafluoroisopropanol (HFIP) to maintain the protecting groups on the amino acid side chains [6]. Peptides were cleaved using a solution 3:1 DCM:HFIP for 2 hours at room temperature. All peptides were reverse phase HPLC purified and lyophilized prior to use.

**General Procedure A**: Amide bond Coupling. The coupling reagent (1-[Bis(dimethylamino)methylene] -1H-1,2,3-triazolo [4,5-b]pyridinium 3-oxide hexafluorophosphate (HATU) was dissolved with the carboxylic acid starting material and 2,4,6-collidene in DMF. The solution of activated acid was added to the amine and agitated at RT.

**General Procedure B**: Allyl Deprotection. $Pd(OAc)_2$ (0.5 equiv.) was mixed with triphenylphosphine ($PPh_3$, 1 equiv) in dry THF under an inert atmosphere of Argon. The solution of activated Pd0 was added to the allyl starting material (1 equiv.) under an inert atmosphere of Argon. Then, phenylsilane ($SiPhH_3$, 5 equiv.) was added to the solution and stirred at RT for 16 h. After the reaction, the solution was concentrated in vacuo and dissolved in 1:1 MeCN:H2O (0.1% TFA) for purification via reverse phase chromatography.

**General Procedure C**: Boc and tBu peptide sidechain deprotection. After the respective amide bond coupling and HPLC purification, the purified product was collected and concentrated in vacuum. The product, was then dissolved 7:2:0.5:0.5 TFA:DCM:H2O:TIS and stirred at RT for 2 h. The reaction was then concentrated in vacuo. The residue was dissolved in 1:1 MeCN:H2O (0.1% TFA) and lyophilized.

**General procedure for loading Fmoc-diaminohexane onto 2-chlorortrityl chloride resin**

256 mg (1 equiv.) of 2-chlorortrityl chloride resin (0.92 mmol/g loading) was weighed into a solid phase reaction vessel. Dichloromethane (DCM) was added to suspend the resin and the vessel was agitated for 5 minutes using a laboratory shaker and the solvent subsequently removed. To a separate flask (9H-fluoren-9-yl)methyl (6-aminohexyl)carbamate (398 mg, 1.18mmol, 5 equiv.),

7mL of DMF, and Diisopropylthylamine (DIPEA) (409uL, 2.35 mmol, 10 equiv.) was added with stir bar. This suspension was heated to 40C and stirred until fully dissolved. Once immediately dissolved the solution was added directly to the 2-chlorortrityl chloride resin. The reaction vessel was capped and agitated for 24 hours at room temperature. The resin was washed DCM (3X), and DMF (3X). The resin was suspended in MeOH for 10 min to deactivate any unreacted trityl resin. The resin was washed DCM (3X), DMF (3X), DCM (1X), dried, and used for synthesis of intermediate 1 (Cat substrate) and intermediate 4 (Casp3 substrate).

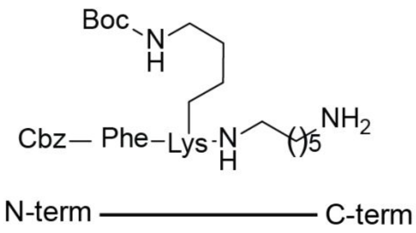


Intermediate 1 (I1) Cat Substrate)

**Intermediate 1**: Intermediate one was synthesized using 2-chlorortrityl chloride resin loaded with Fmoc-diaminohexane with the standard conditions stated in solid phase peptide synthesis.

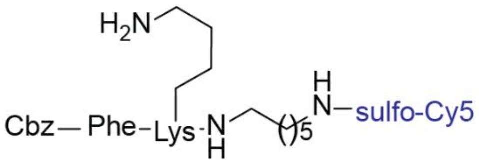


Intermediate 2 (I2) (Cat Substrate)

**Intermediate 2**: I1 (Cat substrate) (8.4mg, 13 umol, 1 equiv.) was dissolved in DMF (1000 µL) with stir bar. Then, DIPEA (24 µL, 0.13 mmol, 10 equiv.) was added and the reaction was stirred for 1 min at RT. Then sulfo-Cy5-Osu (12 mg, 16 umol 1.2 equiv.) was added and allowed to stir for 24 h at RT. The reaction was quenched with the addition of 1:1 MeCN:H2O (0.1% TFA) and purified using a reverse phase Combiflash with a gradient of 5% MeCN:H2O (0.1% TFA) for 0-2 min, 2-95% for 2-18 min, 95% for 18-20 min (Rt = 6 min). The purified fractions were collected and lyophilized to obtain a blue powder (10.9 mg, 65% yield). The purified fractions were collected and General Procedure C was followed to obtain a blue powder.

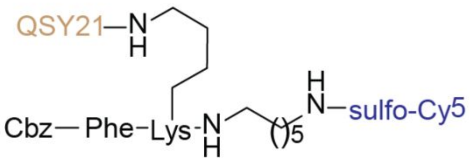


6QC-Cy5

**6QC-Cy5**: See Ofori et. al. or **6QC-RATIO** procedure below.

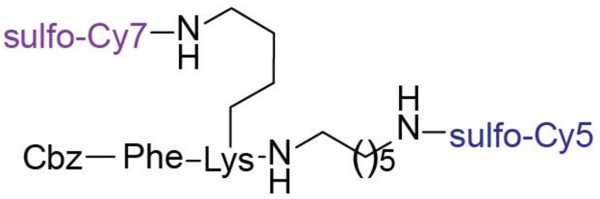


6QC-RATIO

**6QC-RATIO**: I2 (6.0 mg, 5.2 umol, 1 equiv.) was dissolved in DMF (1000 µL) with stir bar. Then, DIPEA (9.1 µL, 52 umol, 10 equiv.) was added and the reaction was stirred for 1 min at RT. Then sulfo-Cy7-Osu (5.5 mg, 6.5umol 1.25 equiv.) was added and allowed to stir for 24 h at RT. The reaction was quenched with the addition of 1:1 MeCN:H2O (0.1% TFA) and purified using a reverse phase Combiflash with a gradient of 5% MeCN:H2O (0.1% TFA) for 0-2 min, 2-95% for 2-18 min, 95% for 18-20 min (Rt = 7 min). The purified fractions were collected and lyophilized to obtain a blue powder (7.1mg, 74% yield). HRMS (ESI$^-$) calculated for $C_{98}H_{119}N_9O_{18}S_4^{2-}$: 919.3800, found 919.3821.

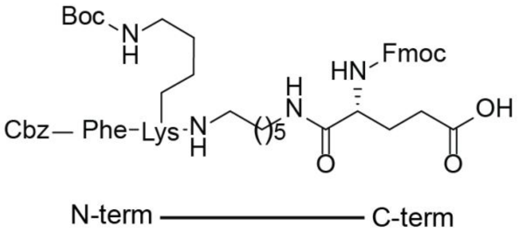


Intermediate 3 (I3)

**Intermediate 3:** Following General Procedure A, Intermediate 1 (30 mg, 48 umol, 1 equiv.), Fmoc-Glu(OAll)-OH (59 mg, .14 mmol, 3 equiv.), HATU (55 mg, 0.14 mmol, 3 equiv.), 2,4,6-collidene (57 µL, 0.43 mmol, 9 equiv.), DMF (1 mL). The reaction was stirred for 16 h and then concentrated in vacuo. The products were dissolved in 7:3 MeCN:H2O (0.1% TFA) and purified using a reverse phase Combiflash with a gradient program of 10% MeCN:H2O (0.1% TFA) for 0-2 min, 10-80% for 2-19 min (Rt = 15.5 min). The purified fractions were collected and lyophilized to obtain a white powder (22 mg, 45% yield). This procedure was repeated multiple times. Next, General Procedure B was used with the substrate, glutamic acid (113 mg, 111 umol, 1 equiv.), Pd(OAc)2 (12.5 mg, 55.5 umol, 0.5 equiv. ), PPh3 (29.1 mg, 111 umol, 1 equiv.), PhSiH3 (68.5 µL, 555 umol, 5 equiv.), THF (5 mL). The reaction was purified using a reverse phase Combiflash with a gradient of 10% MeCN:H2O (0.1% TFA) for 0-2 min, 10- 80% for 2-19 min, 80-95% for 19-25 min (Rt = 16 min). The purified fractions were collected and lyophilized to obtain a white powder (64 mg, 59% yield).

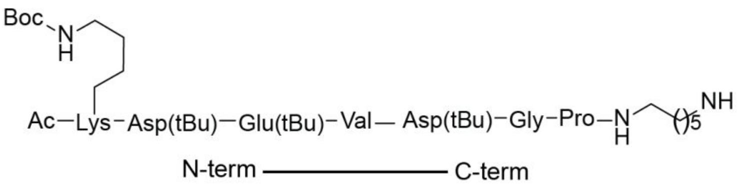

Intermediate 4 (I4) Casp3 Substrate)

**Intermediate 4**: Intermediate 4 was synthesized using 2-chlorortrityl chloride resin loaded with Fmoc-diaminohexane with the standard conditions stated in solid phase peptide synthesis.

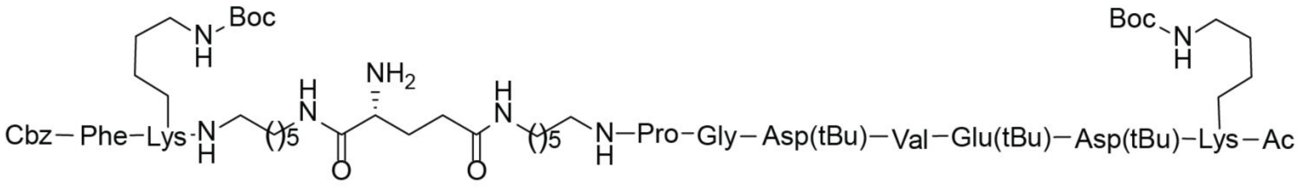

Intermediate 4.5

**Intermediate 4.5**: Following General Procedure A, with Intermediate 3 (52 mg, 53 umol, 1 equiv,), Intermediate 4 (Casp3 substrate) (62 mg, 53 umol, 1 equiv.), HATU (20 mg, 53 umol, 1 equiv.), 2,4,6-collidene (20 µL, 0.16 mmol, 3 equiv.), DMF (1000 µL). The reaction was stirred for 16 h followed by addition of piperidine (200 µL) and then agitated for an additional 1 h. Then, the reaction was concentrated in vacuo. The reaction was dissolved in 1:1 MeCN:H2O (0.1% TFA) and purified using a reverse phase Combiflash (4 g column) with a gradient program of 10% MeCN:H2O (0.1% TFA) for 0-2 min, 10-70% for 2-20 min (Rt = 17.2 min). The purified fractions were collected and lyophilized to obtain a white powder (52 mg, 51% yield).

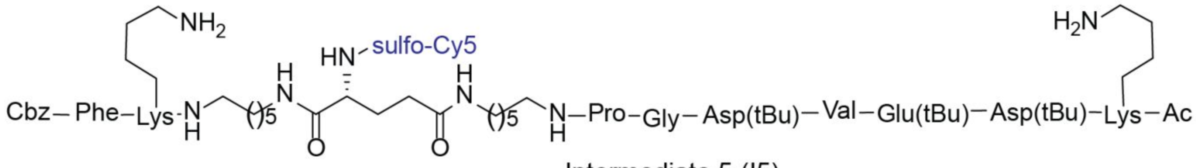

Intermediate 5 (I5)

**Intermediate 5**: Intermediate 4.5 (24 mg, 13umol, 1 equiv.) and sulfo-Cy5-OSu (11 mg, 15umol, 1.2 equiv.) were dissolved in DMF (1000 µL). Then, DIPEA (22 µL, 0.13mmol, 10 equiv.) was added and the reaction was agitated for 24 h at RT. The reaction was quenched with the addition of 1:1 MeCN:H2O (0.1% TFA) and a reverse phase Combiflash with a gradient program of 10% MeCN:H2O (0.1% TFA) for 0-2 min, 10-95% for 2-23 min, 95% for 23-26 min (Rt = 18.6 min). The purified fractions were collected to give a blue powder (13.2 mg, 41% yield) and General Procedure C was followed to obtain a blue powder.

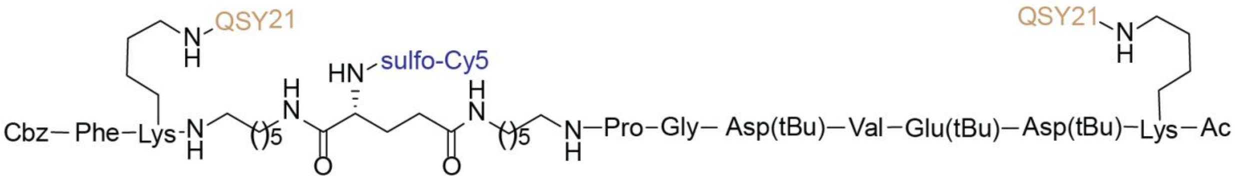


Death-Cat-2

**Death-Cat-2**: See Widen et. al. or **Death-Cat-RATIO** procedure below.

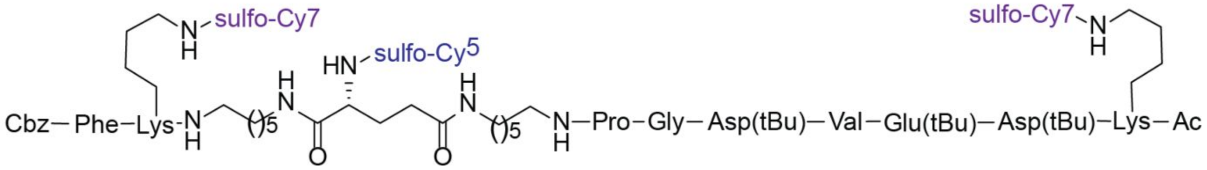


Death-Cat-RATIO

**Death-Cat-RATIO** Intermediate 5 (6.5 mg, 3umol, 1 equiv.) was combined with sulfo-Cy7-OSu (8.2 mg, 9.7 umol, 3.2 equiv.) and then dissolved in DMSO (1000 µL). Then, DIPEA was added (5.2 µL, 30 umol, 10 equiv.) and the reaction was agitated for 24 h at 37 °C. The reaction was quenched by the addition of 1:1 MeCN:H2O (0.1% TFA) and purified with a reverse phase Combiflash with a gradient program of 5% MeCN:H2O (0.1% TFA) for 0-2 min, 5-40% for 2-32 min, 40-95% for 32-36 min (Rt = 24 min). Purified fractions were collected and lyophilized to obtain a blue powder (7.8 mg, 73% yield). HRMS (ESI$^-$) calculated for $C_{179}H_{231}N_{22}O_{41}S_6^{3-}$ $[M-6]^{3-}$: 1179.1680 ; found 1179.1704.

## HPLC Purity Analysis

**6QC-RATIO**

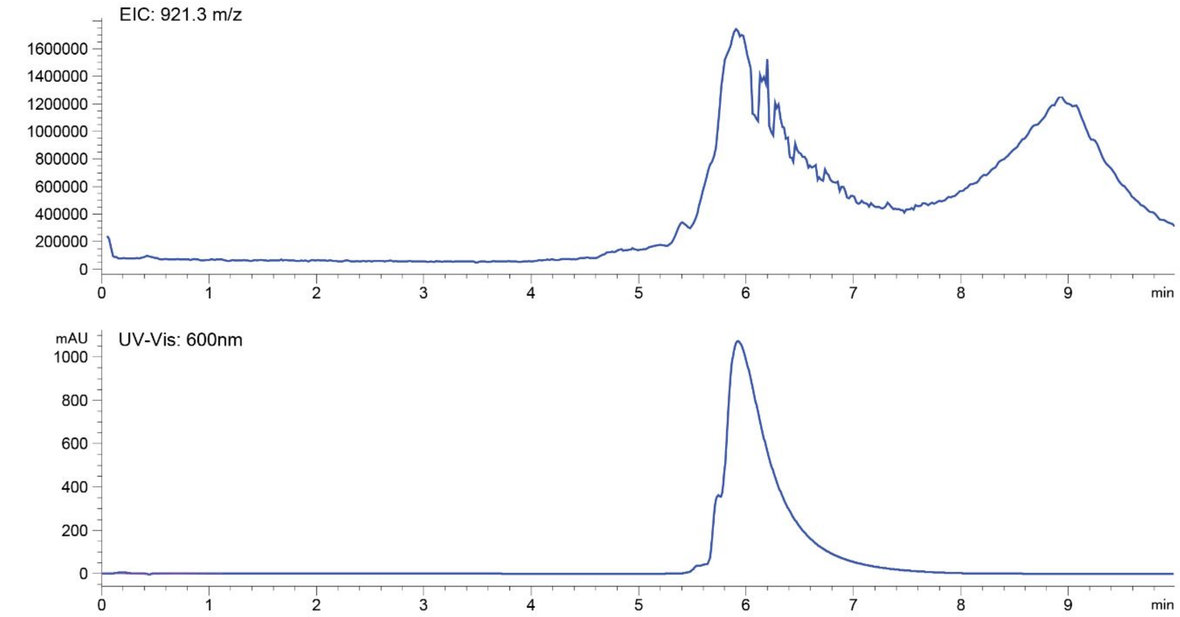


**Death-Cat-RATIO**

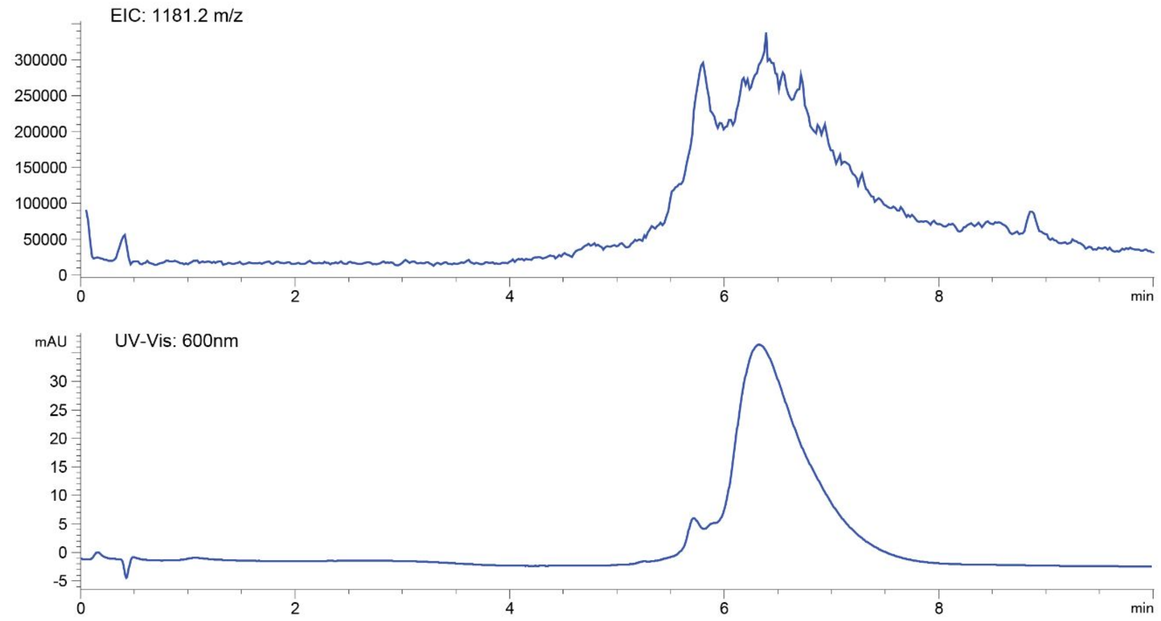

## 6QC-RATIO

FaucherF_12874_FFF2_inf_001 01/20/23 16:03:21

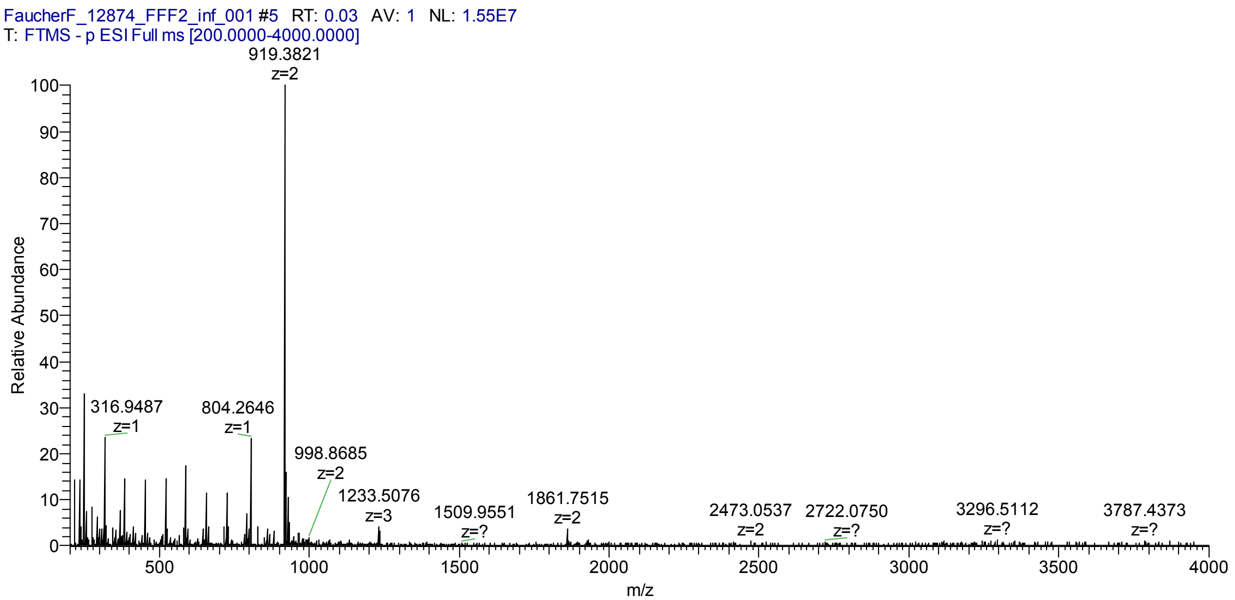


## Death-Cat-RATIO

FaucherF_12874_FFF1_inf_001 01/20/23 16:24:40

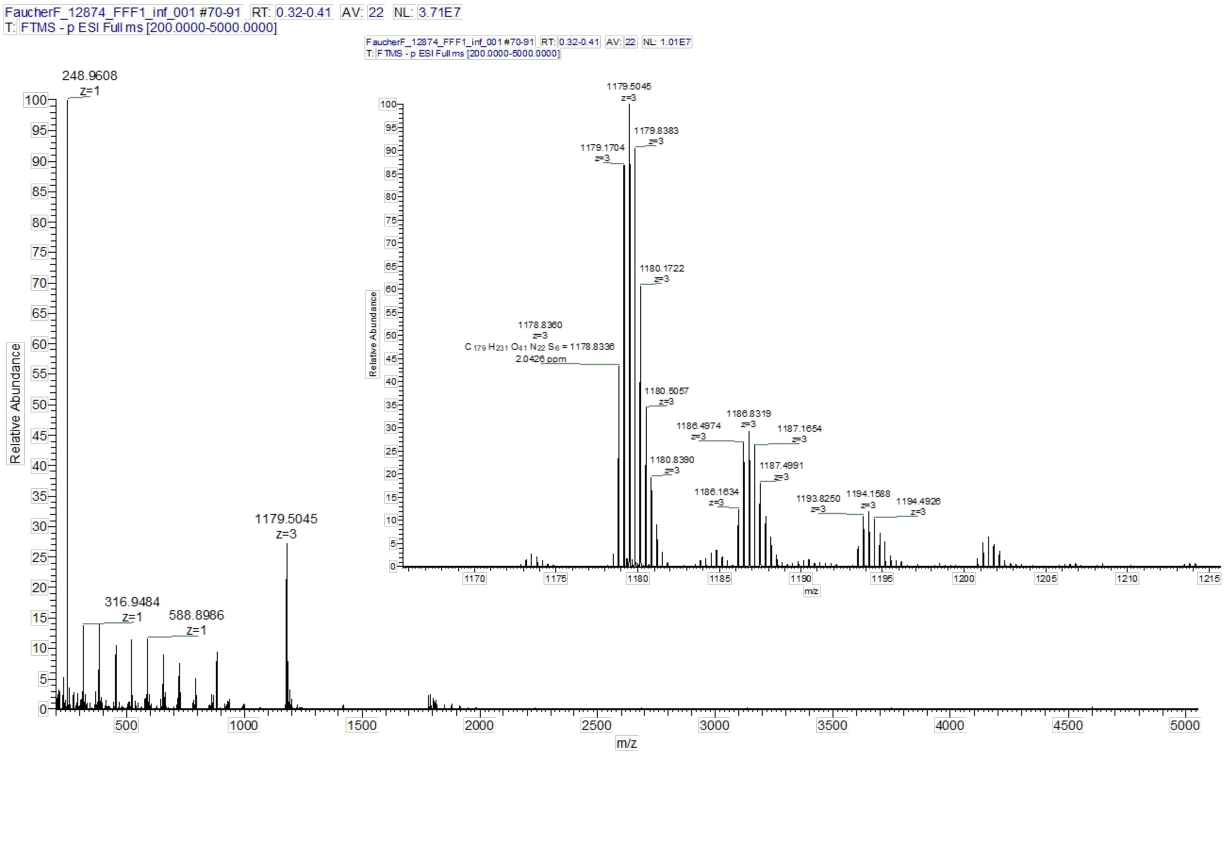

**Animal Experiment Statement**

All experiments performed on live animals were performed in accordance with all national and local guidelines and regulations. All experiments were approved by the APLAC committee at Stanford University.

**Risk Statement**

No unexpected or unusually high safety hazards were encountered.

**References**


(1) Robinson, K. M.; Beckman, J. S. Synthesis of Peroxynitrite from Nitrite and Hydrogen Peroxide. *Methods Enzymol.* **2005**, *396* (05), 207–214. https://doi.org/10.1016/S0076-6879(05)96019-9.

(2) Chen, S.; Vurusaner, B.; Pena, S.; Thu, C. T.; Mahal, L. K.; Fisher, E. A.; Canary, J. W. Two-Photon, Ratiometric, Quantitative Fluorescent Probe Reveals Fluctuation of Peroxynitrite Regulated by Arginase 1. *Anal. Chem.* **2021**, *93* (29), 10090–10098. https://doi.org/10.1021/acs.analchem.1c00911.

(3) Pacher, P.; Beckman, J. S.; Liaudet, L. {suggests Cell Models with Genetic Deletion of INOS as Control Experiment. Can We Do This Too?] Nitric Oxide and Peroxynitrite in Health and Disease. *Physiol. Rev.* **2007**, *87* (1), 315–424.

(4) Widen, J. C.; Tholen, M.; Yim, J. J.; Antaris, A.; Casey, K. M.; Rogalla, S.; Klaassen, A.; Sorger, J.; Bogyo, M. AND-Gate Contrast Agents for Enhanced Fluorescence-Guided Surgery. *Nat. Biomed. Eng.* **2021**, *5* (3), 264–277. https://doi.org/10.1038/s41551-020-00616-6.

(5) Ofori, L. O.; Withana, N. P.; Prestwood, T. R.; Verdoes, M.; Brady, J. J.; Winslow, M. M.; Sorger, J.; Bogyo, M. Design of Protease Activated Optical Contrast Agents That Exploit a Latent Lysosomotropic Effect for Use in Fluorescence-Guided Surgery. *ACS Chem. Biol.* **2015**, *10* (9), 1977–1988. https://doi.org/10.1021/acschembio.5b00205.

(6) Bollhagen, R.; Schmiedberger, M.; Barlos, K.; Grell, E. A New Reagent for the Cleavage of Fully Protected Peptides Synthesised on 2-Chlorotrityl Chloride Resin. *J. Chem. Soc., Chem. Commun.* **1994**, 2559.
