# Supplementary material for: Protease Activated Probes for Real-Time Ratiometric Imaging of Solid Tumors
Source: ACS Cent Sci. 2023 May 4;9(5):1059–69. doi: 10.1021/acscentsci.3c00261 (PMC10214504; doi:10.1021/acscentsci.3c00261)
Supplement: Supplementary file 4 — oc3c00261_si_004.pdf [file oc3c00261_si_004.pdf]

oc-2023-00261k.R1

Name: Peer Review Information for "Protease activated probes for real-time ratiometric imaging of solid tumors"

First Round of Reviewer Comments

Reviewer: 1

Comments to the Author

This manuscript describes the development of ratiometric strategy to provide protease activatable probes for use in optically guided surgical procedures. Building upon prior work from this group, which has led to agents with significant potential for clinical use, the author details the advantages of a ratiometric version of their approach (previously a quenched probe strategy). In the strategy reported here, the “Off-State” signal can be read out on a Cy7 channel (via FRET), whereas the “ON” state is imaged on the the Cy5 channel. This allows both states to be measured concurrently, allowing for ratiometric imaging The authors create the probes and the imaging to test this approach, which is validated through in vitro and in vivo testing. This approach provides a means to converts fluorescent quenched probes, which have been used extensively by the protease community, into ratiometric optical contrast agents. The work is quite creative and will be of interest to a broad range of groups working in this area and beyond. The minor comments below should be addressed if possible.

1. The authors should provide more detailed characterization of the FRET transfer process, as various efforts in this context have seen only modest transfer to certain acceptor due to quenching effects. Ideally using fluorimeter-type studies to investigate the degree of FRET transfer, or at least showing the relative magnitude of the signal in the imaging system.

2. The author should discuss in more detail why they believe this strategy improves the signal in the liver, specifically the comparison of the Cy5 channel between the two probes in 4e. Prior work has shown a key role for macrophage uptake in the liver. Given that, why should one expect that the ratiometic strategy reduces the amount of Cy5 signal, vs. not just improves the ratioed signal?

Minor comments

1. The authors might consider moving some of the supplementary figures into the main text. For example the data in supp figure 7 reveals a neat aspect of this strategy.

2. The cell line used in vitro testing could only be found in the supplementary information, even though that is information that would be of interest to many readers.

Reviewer: 2

Comments to the Author

The manuscript under consideration by Bogyo and coworkers describes a simple yet effective strategy to convert conventional protease probes that utilize the FRET mechanism to achieve a turn-on response, into ratiometric sensor system for improved performance. Specifically, the authors replaced the critical dark quencher component with a second fluorophore (Cy7) to enable this technology. Beyond the conversion of examples such as 6QC-Cy5 to 6QC-RATIO, the authors also developed a dual-substrate AND-gate ratiometric probe using this strategy (i.e., DeathCat-RATIO). A notable advance is that an imaging system that could be coupled to the FDA-approved da Vinci Xi® robot was also developed in this study, which enabled real-time imaging of ratiometric signals. Together, the authors overcame a key limitation associated with the application of probes fluorescence-guided surgery, which artifacts that arise from incorrect positioning of the camera setup. Overall, this paper should be suitable for publication after the following concerns are addressed.

1. Longer cyanines such as Cy7 are prone to oxidative cleavage by RNOS such as ONOO-; however, there were no stability assays to demonstrate this. This experiment should be performed.

2. The authors should vary the amount (activity) of cathepsin L to show dose-dependency.

3. Not all ratiometric systems involves the increase of one signal with the simultaneously decrease in the other. Indeed, there are designs where the intensity of one component remains consistent. This should be briefly mentioned.

4. What are the average size of the distal tumors vs the in-focus tumors. Did the authors flip the position of the animals such that the in-focus tumor becomes the distal tumor?

Minor typos

1. surgery(3) radiation – missing comma

Author's Response to Peer Review Comments:

Response to Reviewers

We thank the reviewers for their positive evaluations and insightful suggestions. We would like to thank the editor for seeing the value of this work to the broader community and the reviewers for recommending this work for a First Reactions (a News & Views piece in the journal). We have addressed all comments and suggestions in our revised manuscript and the point-by-point response below. We have highlighted changes in the text to reflect these revisions.

Formatting Needs:

We have completed all formatting requests and highlighted these changes.

Reviewer: 1

Comments:

This manuscript describes the development of ratiometric strategy to provide protease activatable probes for use in optically guided surgical procedures. Building upon prior work from this group, which has led to agents with significant potential for clinical use, the author details the advantages of a ratiometric version of their approach (previously a quenched probe strategy). In the strategy reported here, the "Off-State" signal can be read out on a Cy7 channel (via FRET), whereas the "ON" state is imaged on the Cy5 channel. This allows both states to be measured concurrently, allowing for ratiometric imaging The authors create the probes and the imaging to test this approach, which is validated through in vitro and in vivo testing. This approach provides a means to converts fluorescent quenched probes, which have been used extensively by the protease community, into ratiometric optical contrast agents. The work is quite creative and will be of interest to a broad range of groups working in this area and beyond. The minor comments below should be addressed if possible.

1. The authors should provide more detailed characterization of the FRET transfer process, as various efforts in this context have seen only modest transfer to certain acceptor due to quenching effects. Ideally using fluorimeter-type studies to investigate the degree of FRET transfer, or at least showing the relative magnitude of the signal in the imaging system.

We have added Supplementary Figure 1 where we characterize the FRET transfer of our probes. Specifically, we show dose dependent cleavage of our FRET probe upon addition of cathepsin L. Furthermore, we have calculated the fold change in donor fluorescence emission upon cleavage by cathepsin L using the signal from the no probe baseline.

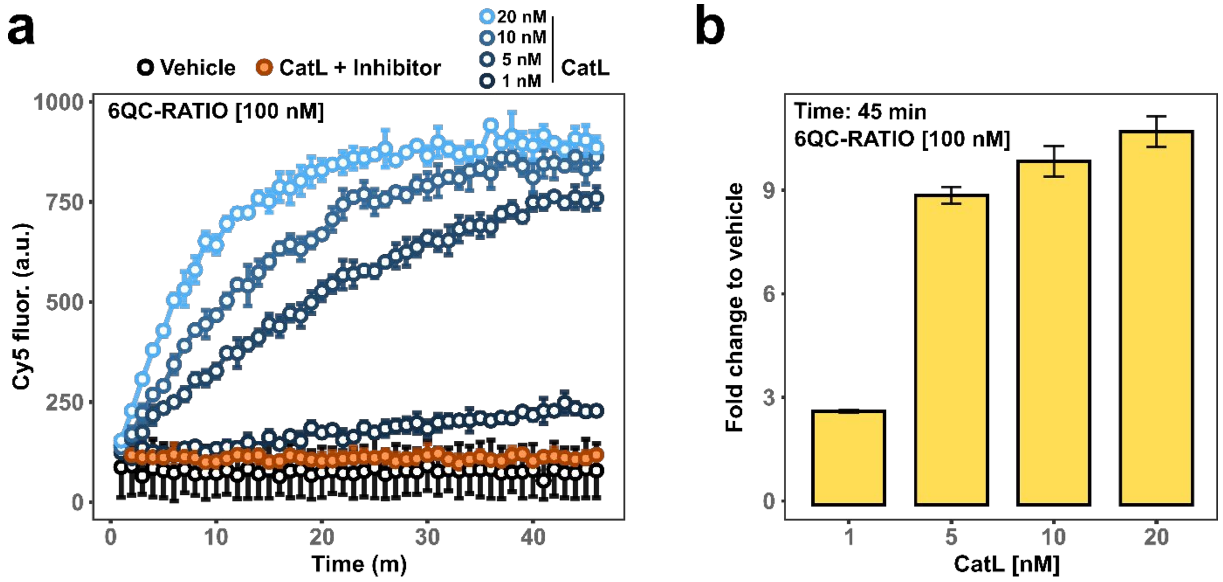

Supplementarty figure 1. In vitro *characterization of FRET performance upon cathepin L cleavage.* ***A)*** *Fluorimetric time course analysis of 6QC-RATIO cleavage upon cathepsin L addition (at 1, 5, 10 or 20 nM concentration), vehicle addition or addition of cathepsin L [10 nM] preincubated 30 min with a cathepsin inhibitor E64d* [10 µM]. ***B)*** *Bar graph showing the fold change in Cy5 fluorescence emission 45 min after addition of cathepsin L showing dequenching of Cy5 upon cathepsin processing. Data are shown as mean ± sd of three technical replicates.*

2. The author should discuss in more detail why they believe this strategy improves the signal in the liver, specifically the comparison of the Cy5 channel between the two probes in 4e. Prior work has shown a key role for macrophage uptake in the liver. Given that, why should one expect that the ratiometic strategy reduces the amount of Cy5 signal, vs. not just improves the ratioed signal?

We agree with the reviewer that the ratiometric strategy should not reduce the amount of Cy5 signal that accumulates in the liver due to macrophage uptake. In fact, our strategy improves only the ratioed signal as the reviewer suggested. The difference in Cy5 signal shown in figure 4e derives from an adjustment in the histograms that we performed for visualization purposes and not from an actual different accumulation of the probes in the liver. WE mistakenly did not include the intensity scale for the Death-Cat probe so it appeared that the images were on the same scale. The image below shows the raw signals for the two probes set to the same scale. To fix this errot, we have updated the figure 4e so that the image now shows all the cy5 figures on the same histogram range. This image clearly shows that there is no difference in overall liver accumulation for the Ratio probe compared to the quenched probe.

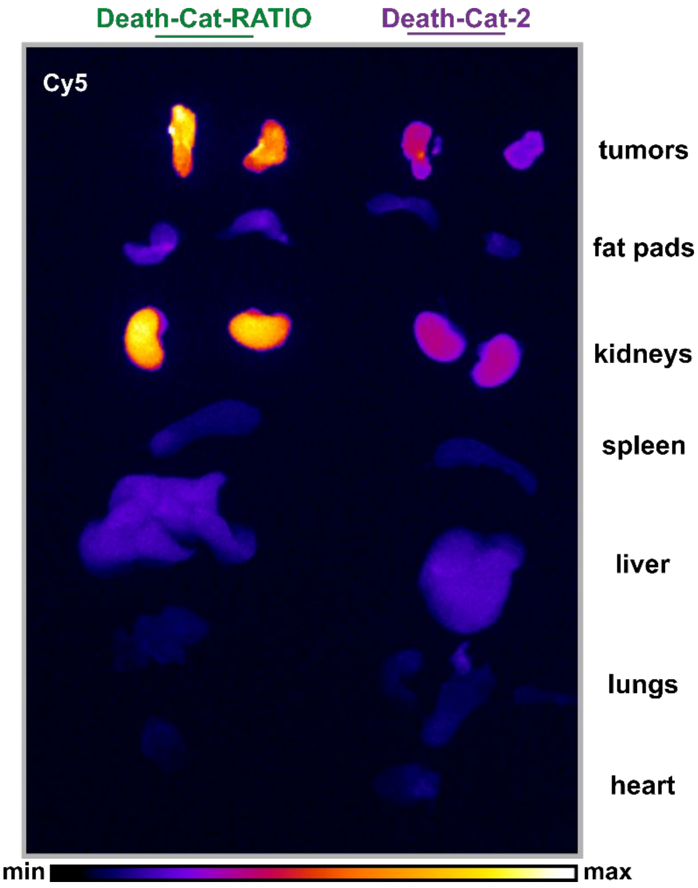

Minor comments

1. The authors might consider moving some of the supplementary figures into the main text. For example the data in supp figure 7 reveals a neat aspect of this strategy.

We have added the supplementary figure 7 as part of the main text and it is now shown as figure 6 c and d.

2. The cell line used in vitro testing could only be found in the supplementary information, even though that is information that would be of interest to many readers.

We have added both cell lines and their product number used in this manuscript 4T1 (ATCC CRL-2539) and RAW 264.7 (ATCC TIB-71) to the main text and have updated the supplementary with the product number.

Reviewer: 2

Comments:

The manuscript under consideration by Bogyo and coworkers describes a simple yet effective strategy to convert conventional protease probes that utilize the FRET mechanism to achieve a turn-on response, into ratiometric sensor system for improved performance. Specifically, the authors replaced the critical dark quencher component with a second fluorophore (Cy7) to enable this technology. Beyond the conversion of examples such as 6QC-Cy5 to 6QC-RATIO, the authors also developed a dual-substrate AND-gate ratiometric probe using this strategy (i.e., DeathCat-RATIO). A notable advance is that an imaging system that could be coupled to the FDA-approved da Vinci Xi® robot was also developed in this study, which enabled real-time imaging of ratiometric signals. Together, the authors overcame a key limitation associated with the application of probes fluorescence-guided surgery, which artifacts that arise from incorrect positioning of the camera setup. Overall, this paper should be suitable for publication after the following concerns are addressed.

1. Longer cyanines such as Cy7 are prone to oxidative cleavage by RNOS such as ONOO-; however, there were no stability assays to demonstrate this. This experiment should be performed.

We agree that testing the oxidative cleavage of our probe valuable for understanding the stability of our probe *in vivo*. To address this, we have incubated 6QC-RATIO and Death-Cat-RATIO with physiologically relevant concentrations of peroxynitrite 0-200 nM, and higher (Figure S5). As expected, peroxynitrite does reduce the absorbance of these probes indicating breakdown of the cyanine dyes. However, the reduction in absorbance is less than 50% for both wavelengths at concentrations of 200nM Peroxynitrite, which is on the higher end of potential physiological concentration of RNOS. We also observed that Cy7 absorbance was impacted more than Cy5 as expected since longer cyanines are more prone to oxidative cleavage. Overall, this data agrees with the expected oxidative cleavage of cyanines by RNOS. We believe that the overall degradation iby RNOS in vivo is likely to be quite minimal such that it is unlikely to impact the overall utility of these probes in vivo. We have discussed these findings in the main text and have added this new data to supplementary Figure S5.

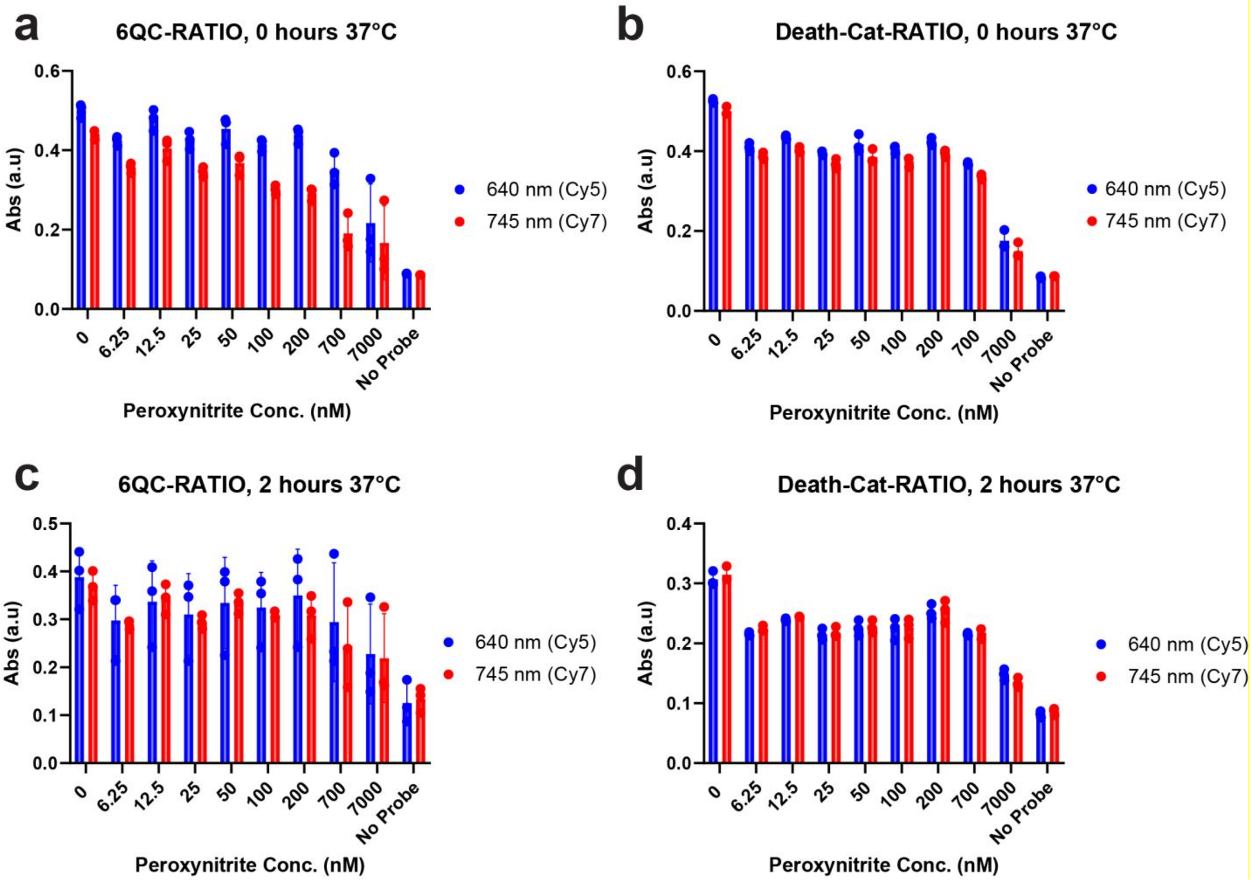

Figure S5. Degradation assay of cyanine dyes with freshly generated peroxynitrite a reactive nitrogen oxide species. 6QC-RATIO or Death-Cat-RATIO at 7uM (physiological concentration) were incubated with peroxynitrite and absorbance was measured at both 640 nm and 745 nm. Time points were taken immediately after addition and 2 hours after incubating at 37° C. A) Absorbance for 6QC-RATIO at 0 hours. B) Absorbance for Death-Cat-RATIO at 0 hours. C) Absorbance for 6QC-RATIO at 2 hours. D) Absorbance for Death-Cat-RATIO at 2 hours. Data are shown as mean ± sd of three technical replicates.

2. The authors should vary the amount (activity) of cathepsin L to show dose-dependency.

In our response to reviewer 1, we have detailed cathepsin L dose dependence and included additional FRET characterization. This new data can be found in Supplementary figure 1.

3. Not all ratiometric systems involves the increase of one signal with the simultaneously decrease in the other. Indeed, there are designs where the intensity of one component remains consistent. This should be briefly mentioned.

We agree with the reviewer that there are other ratiometric systems of value that should be discussed. In paragraph seven of the introduction, we have detailed ratiometric probe design where the intensity of one reference dye remains consistent while the other is activated. We have added the relevant citation for this design in the highlighted section and included reference 34.

4. What are the average size of the distal tumors vs the in-focus tumors. Did the authors flip the position of the animals such that the in-focus tumor becomes the distal tumor?

The average size of the distal tumors and the in-focus tumors have been included for the 4T1 model in Supplementary Figure 6b. The position of the animals was exchanged during the imaging such that each tumor was imaged as both the in-focus proximal tumor and out of focus distal tumor.

<u>Minor typos</u>

1. surgery(3) radiation – missing comma

We made this edit.
